# Genetic Analyses of Flower, Fruit, and Stem Traits of Intergeneric Hybrids Between ‘Honghuagqinglong’ and ‘Heilong’ Pitayas

**DOI:** 10.3390/plants13243546

**Published:** 2024-12-19

**Authors:** Xinyue Pu, Imran Khan, Tiantian Zhang, Guohua Huang, Jiayi Chen, Yu Ding, Xuewu Ji, Zhike Zhang, Jietang Zhao, Guibing Hu, Irfan Ali Sabir, Yonghua Qin

**Affiliations:** 1 Key Laboratory of Biology and Genetic Improvement of Horticultural Crops (South China), Ministry of Agriculture and Rural Affairs, College of Horticulture, South China Agricultural University, Guangzhou 510642, China; 13698166417@163.com (X.P.); imran.62k@gmail.com (I.K.); 17863608557@163.com (T.Z.); chenjiayi98@stu.scau.edu.cn (J.C.); d2423572769@163.com (Y.D.); jxw411099230@163.com (X.J.); poloky2@163.com (Z.Z.); zhaojietang@gmail.com (J.Z.); guibing@scau.edu.cn (G.H.); 2 Inner Mongolia Xuandatai Agricultural Technology Co., Ltd., Hohhot 010010, China; xdtkj2018@163.com; 3 Guangdong Provincial Key Laboratory of Postharvest Science of Fruits and Vegetables, College of Horticulture, South China Agricultural University, Guangzhou 510642, China

**Keywords:** pitaya, intergeneric hybrid, flower traits, fruit traits, stem traits, genetic variation

## Abstract

Pitaya is renowned for its delicious taste, high nutritional value, and economic as well as ornamental appeal. Breeding new pitaya varieties can boost economic returns by appealing to consumers with diverse morphological traits. However, the genetic basis underlying key traits in intergeneric hybrids of pitaya has yet to be fully understood. This study investigates the genetic dynamics in flower, fruit, and stem traits, including segregation patterns and a mixed inheritance model for major and polygenic traits, in an intergeneric hybridization between ‘Honghuagqinglong’ (HHQL) (*Hylocereus stenopterus*) and ‘Heilong’ (HL) (*Selenicereus grandiflorus*). The study identified normal or skewed, normal distribution patterns in seven floral, fifteen fruit, and five stem traits, indicating their quantitative nature governed by multiple genes. Specifically, flower size and color exhibited a hereditary bias towards ‘HL’ characteristics, while ‘HHQL’ significantly influenced the coloration of fruit peel and pulp. Fruit weight and total soluble solids (TSS) content decreased, whereas stem traits exhibited broader and thicker dimensions with fewer thorns. This study offers valuable insights into genetic variation and the influence of major genes on flower, fruit, and stem traits between ‘HHQL’ and ‘HL’ intergeneric hybrids, providing a useful reference for parental selection in pitaya breeding programs.

## 1. Introduction

Pitaya is one of the delicious and tropical fruits belonging to the genus *Hylocereus* (nested within *Selenicereus*) within the Cactaceae and originated from Mexico, Central America, and South America [1,2,3]. Pitaya has grown into a globally cultivated fruit, encompassing a wide range of species suited to various climates. Known for its sweet flavor and medicinal qualities, pitaya is rich in plant-based nutrients that contribute to its health benefits. This nutrient profile includes carbohydrates, dietary fiber, proteins, vitamins, flavonoids, organic acids, essential minerals, and amino acids, positioning pitaya as a valuable addition to a balanced, nutritious diet [4,5,6]. Pitaya fruit is also abundant in unique components such as betalain, plant-based albumin, and water-soluble dietary fiber. Pitaya flowers can be processed as tea, and seeds are rich in unsaturated fats with high oxidative stability including antioxidant properties, anti-aging benefits, and skin-brightening effects [1,6]. In addition, pitaya is very useful for promoting bowel movement, reducing cholesterol, and preventing colon cancer [7,8].

Pitaya is a resilient crop with substantial economic potential, particularly suited to challenging growing conditions. This tropical fruit is also economically significant, with its cultivation expanding across countries like China, Vietnam, Thailand, the Philippines, Australia, and Israel. Pitaya’s resilience to high temperatures and poor soil conditions further underscores its agricultural potential, making it an increasingly popular choice among farmers. Moreover, its ornamental qualities, alongside its health benefits, contribute to its high market demand and economic value. It can withstand high temperatures up to 40 °C and thrives in dry, nutrient-poor soils, making it adaptable to diverse environments [1,2,9]. This resilience has contributed to its popularity among farmers and home gardeners in numerous countries, including China, Vietnam, Thailand, Malaysia, Indonesia, the Philippines, Bangladesh, Australia, the United States, and Israel. With promising prospects for production, trade, and consumption, pitaya is becoming an economically valuable crop with a strong market demand [10].

Genetic composition and improvements in plant traits play critical roles in determining fruit quality, yield, and shelf life. At present, two primary types of pitaya white-flesh (*H. undatus*) and red-flesh (*H. monacanthus*) are widely cultivated on a commercial scale. In recent years, significant efforts in cross-breeding have led to the development of new pitaya varieties, each selected for desirable traits aimed at meeting market demands [11,12], seedling selection [13,14,15,16,17,18], and bud mutation selection [19,20,21]. Elucidation of the genetic laws of traits is an important foundation for fruit breeding that has been reported in longan [22], apple [23], mango [24], and cowberry [25]. Flowers play a vital role as the reproductive organs of plants, and their genetic traits are key to successful pollination and fruit development. Recently, there has been increasing interest in understanding the genetic basis of flower traits in fruit trees, driven by the diversity in phenotypic characteristics across various species. Studies have highlighted the extensive phenotypic variation in flower traits within germplasm resources of several fruit trees, including pear, which holds potential for improving breeding strategies and enhancing crop resilience [26], apricot [27], olive [28], cherry [29], and jujube [30].

Pitaya is valued not only for its nutritional benefits but also for its ornamental appeal, offering promising market potential. The varieties currently available in the market commonly feature white flowers, red peel, and red or white pulp. However, the genetic basis of critical traits in intergeneric hybrid populations of pitaya remains insufficiently understood. Recent advancements in genetic studies and breeding techniques have focused on improving pitaya varieties to meet consumer preferences and market demands. These efforts include enhancing fruit quality, increasing yield, and introducing new traits such as unique peel and pulp colors, early flowering, and disease resistance. Despite progress, the genetic basis of critical traits, particularly in intergeneric hybrids, remains insufficiently understood. Addressing this gap, the present study investigates the genetic dynamics of key flower, fruit, and stem traits using hybrids between *H. stenopterus* (‘Honghuaqinglong’) and *S. grandiflorus* (‘Heilong’). By analyzing the inheritance patterns and quantitative nature of these traits, this research aims to provide valuable insights for the development of improved pitaya germplasm, thereby advancing both the scientific understanding and economic potential of this crop.

## 2. Materials and Methods

### 2.1. Plant Materials

‘Guanhuahong’ (*H. monacanthus*), ‘Honghuaqinglong’ (HHQL) (*H. stenopterus*), ‘Heilong’ (HL), and (*S. grandiflorus*), F_1_ progenies of 150 ‘HHQL’ × ‘HL’ and 100 ‘HL’ × ‘HHQL’ cross combinations were used as parental materials. ‘HHQL’ × ‘HL’ and ‘HL’ × ‘HHQL’ are regarded as positive and negative cross combinations, respectively. ‘Guanhuahong’ pitaya was used as rootstocks. All materials were planted in the pitaya germplasm resource at the College of Horticulture, South China Agricultural University (113.36° N, 23.11° E), China. Petals were red and white for ‘HHQL’ and ‘HL’ pitayas, respectively (Supplementary Figure S1). The petal numbers of ‘HL’ pitaya were significantly higher than that of ‘HHQL’ pitaya. Compared with ‘HL’ pitaya, stigma pork of ‘HHQL’ pitaya was present. Stigmas of ‘HHQL’ pitaya were significantly higher than anthers while the stigma-anther relative position of ‘HL’ pitaya were equal (Supplementary Figure S1 and Table S1), and both cannot self-pollinate.

In terms of the peel and pulp color, ‘HHQL’ pitaya has a green peel with white pulp, while ‘HL’ pitaya has a red peel with red pulp. The average fruit weight of ‘HHQL’ pitaya was greater than ‘HL’ pitaya. The TSS content of ‘HHQL’ pitaya was significantly higher than ‘HL’ pitaya. The stems of ‘HHQL’ pitaya were wider than that of ‘HL’ pitaya, with thinner edges (Supplementary Table S2). The stems of ‘HL’ pitaya were narrower and fuller, with shorter thorns. Furthermore, our previous studies have shown ‘HL’ pitaya was a disease-resistant germplasm resource.

### 2.2. Acquirement of Hybrids

The stamens of ‘HHQL’ and ‘HL’ pitayas were completely removed at 2:00 p.m. on the day of blossoming. The flowers were completely covered with non-woven cloth bags. Pollen from male plant was collected to pollinate onto the stigma of maternal plant at 10:00 in the night. Flowers were covered with non-woven bags, and the bags were removed at 9:00 a.m. the next morning (Supplementary Figure S2). Pulp from fully mature fruit was collected and put in a nylon mesh bag to get rid of pulp fibers under running water. The seeds were soaked in water and germinated at 28 °C on a shaker at 180 rpm for 48 h. The water was changed every 12 h. Germinated seeds were mixed with nursery soil and evenly sewn in seedling trays. Once the hybrid seedlings reached 6–8 cm in height, they were grafted onto 0.5 m rootstocks of ‘Guanhuahong’ pitaya to promote earlier flowering and fruiting.

### 2.3. Authenticity Identification of Hybrid Offspring

DNA from ‘HHQ’, ‘HL’, and their cross offsprings was extracted using the DNA extraction kit (Aidlab Biotechnologies., Ltd, Beijing, China) according to the manufacturer’s instructions. True hybrid identification was verified using SCoT and SRAP-PCR-based system established by our previous study. Sixteen SCoT primers (Supplementary Table S3), and seventeen pairs of SRAP primers (Supplementary Tables S4 and S5) were used for true hybrid identification. The SCoT-PCR-based reactions were conducted in a 20 μL reaction mixture containing 3.05 μL of template DNA, 2 μL 10× buffer, 2.5 μL MgCl_2_ (25 mM), 1.52 μL dNTPs (2.5 mM), and 5 U/μL *Taq* DNA polymerase. The PCR amplification was performed at 94 °C for 5 min, 94 °C for 50 s, then 56 °C for 1 min, and 72 °C for 2 min, followed by 35 cycles, and the final extensions were done at 72 °C for 10 min. The SRAP-PCR-based reactions were conducted in a 20 μL reaction mixture containing 2 μL of template DNA, 10 μL 2× *Taq* Master Mix, 6 μL ddH_2_O, 1 μL forward primer and 1 μL reverse primer. The PCR process was initiated at 94 °C for 5 min, 94 °C for 1 min, then 35 °C for 1 min, 72 °C for 1.5 min, by 5 cycles, then 94 °C for 1 min, 50 °C for 1 min, 72 °C for 1.5 min, followed by 35 cycles, and the final extensions lasted 10 min at 72 °C for 10 min.

### 2.4. Observation and Measurement of Flower, Fruit, and Stem Traits

Traits of the flowers, fruits, and stems of two parents and their F1 progenies were recorded according to the Guidelines of *Hylocereus* descriptors [31]. Flower length, corolla width, and calyx tube width were measured in the afternoon before the opening of pitaya flower at night. The number of petals, development of stigmas, number of stigmatic lobes, and the distance from stamen to stigma were recorded at about 10:00 in the evening.

Stem width, edge thickness, thorn length, distance between thorns, fruit longitudinal and transverse diameters, top cavity, peel thickness, and width of the base of middle calyx were measured using a digital vernier caliper (Shanghai Manette Industries (Group) Co., Ltd., DEGUQMNT, Shanghai, China). TSS content in the center of fruit pulp was determined using a digital refractometer. The color of peel and pulp was measured using a colorimeter. Pitaya canker disease was investigated according to the Supplementary Table S6.

### 2.5. Determination of Betalain Content in Petal

Petals from parental plants and F1 progenies were collected and ground into powder using a rapid grinder in liquid nitrogen. The extraction of betalain was performed by following the method of Hua et al. [32]. Petal’s color was represented according to the betalain content. 

### 2.6. Inheritance Analyses of Pitaya Main Traits

Inheritance of flower, fruit, and stem main traits were analyzed according to our previous study [33].

### 2.7. Data Analyses

Data of flower, fruit, and stem traits were obtained from 2021–2023. IBM SPSS Statistics 26.0 was used for construction of a normal distribution graph, creation of a frequency distribution histogram, calculation of data correlation, and analyses of significant differences. The principal component analysis (PCA) on the data and the genetic laws of each trait were also calculated. The relevant calculation formulas are as follows:

Coefficient of Variation (*CV*) (%) = S/F × 100,

Heritability (*Ta*) (%) = F/MP × 100,

Dominance Ratio (*Ha*) (%) = (F − MP)/MP × 100.

S is standard deviation, F presents average value of the F_1_ generation, and MP stands for mid-parent value [34].

## 3. Results

### 3.1. Identification of Hybridization Offspring

SCoT and SRAP molecular markers confirmed the authenticity of hybridization in F1 progenies that were randomly selected from the cross combinations of ‘HL’ × ‘HHQL’ and ‘HHQL’ × ‘HL’ pitayas. Four SCoT primers (SCoT-63, SCoT-12, SCoT-42, and SCoT-21) and four pairs of SRAP (SRAP-M14E15, SRAP-M16E13, SRAP-M14E15, and SRAP-M16E13) with stable, highly polymorphic bands were selected for identification of the reciprocal cross combinations. The results showed that all F1 progenies of 150 ‘HHQL’ × ‘HL’ and 100 ‘HL’ × ‘HHQL’ cross combinations were true hybrids. All the identification results are shown in Supplementary Figure S3.

### 3.2. Flower Traits

#### 3.2.1. Petal Color and Stigma Bifurcates Traits

**Figure 1:**
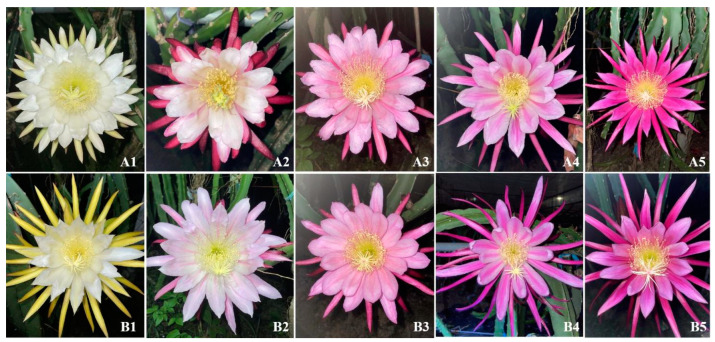
Phenotypes of flower color in F_1_ progenies from ‘HHQL’ × ‘HL’ (**A**) and ‘HL’ × ‘HHQL’ (**B**) cross combinations. 1, white; 2, pale pink; 3, pink; 4, bicolor; 5, red.

**Figure 2:**
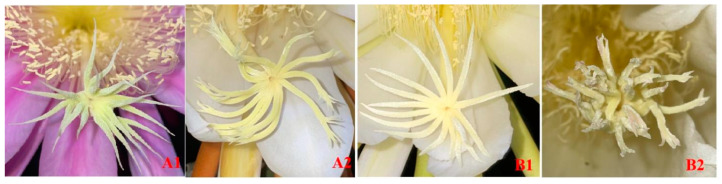
The stigma traits in F_1_ progenies from ‘HHQL’ × ‘HL’ (**A**) and ‘HL’ × ‘HHQL’ (**B**) cross combinations. 1, without bifurcates at the end of stigma; 2, with bifurcates at the end of stigma.

Abundant genetic variations were detected in petal color and stigma bifurcates of F_1_ progenies from cross combinations of ‘HHQL’ × ‘HL’ and ‘HL’ × ‘HHQL’ pitayas (Figure 1 and Figure 2). F1 progenies from ‘HHQL’ × ‘HL’ and ‘HL’ × ‘HHQL’ crosses exhibited distinct segregation patterns in petal color and the largest proportion of petal color was white. There were 51.3%, 4.7%, 13.3%, 2.7%, and 28.0% for white, pale pink, pink, bicolor, and red, respectively, in F1 progenies of ‘HHQL’ × ‘HL’ cross combination when compared with 59.0%, 3.0%, 12.0%, 2.0%, and 24.0% for ‘HL’ × ‘HHQL’. In ‘HHQL’ × ‘HL’ cross combination, 33.3% F1 progenies had stigma bifurcates as compared to 11.3% F1 progenies with stigma bifurcates for ‘HL’ × ‘HHQL’ cross-combination.

#### 3.2.2. Distributions of Main Flower Traits

**Figure 3:**
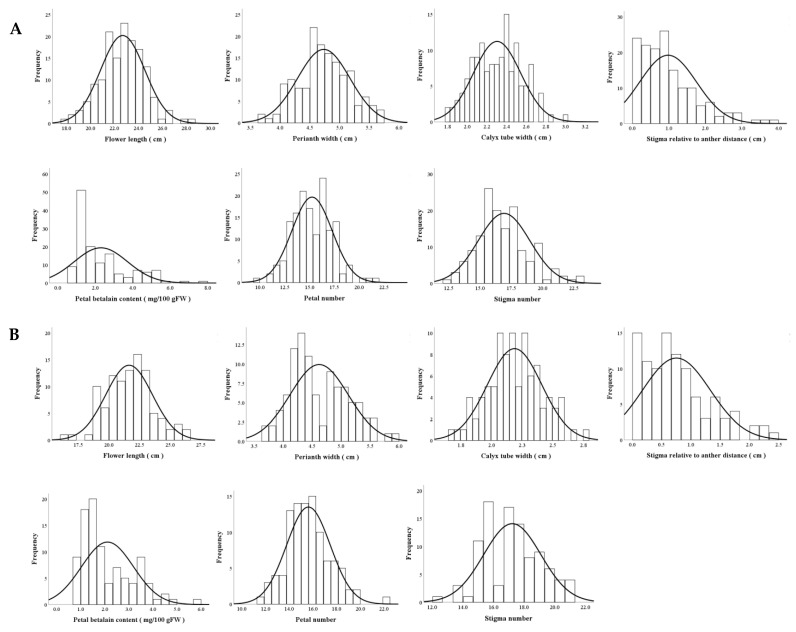
Frequency distribution of ‘HHQL’ × ‘HL’ (**A**) and ‘HL’ × ‘HHQL’ (**B**) cross combinations.

Seven main traits of F_1_ progeny from cross combinations of ‘HHQL’ × ‘HL’ and ‘HL’ × ‘HHQL’ including flower length, perianth width, calyx tube width, stigma relative to anther distance, betalain content in petals, and the number of petal and stigma were analyzed. The phenotypic values for seven flower traits in most offspring fell within the range observed in the parental lines ‘HHQL’ and ‘HL’ pitayas, with some individuals displaying traits that exceeded parental values. As shown in Figure 3, characteristics such as flower length, perianth width, calyx tube width, stigma-to-anther distance, betalain concentration in petals, and the number of petals and stigmas showed either a normal or slightly skewed distribution. These patterns indicate that these seven flower traits are quantitative and likely controlled by multiple genes.

#### 3.2.3. Genetic Analyses of Flower Traits

As shown in Table 1, flower length, perianth width, calyx tube width, distance between stigma and anther, betalain content in petals, number of petals, and stigma lobes had different degrees of variation (Table 1). Flower length, perianth width, calyx tube width, petal, and stigma lobe number exhibited similar patterns of variation. They all had lower coefficients of variation and higher genetic transmission ability, indicating that these traits are less influenced by environmental factors and can be stably inherited.

In the F1 progenies of the ‘HHQL’ × ‘HL’ and ‘HL’ × ‘HHQL’ cross combinations, the coefficients of variation for stigma-to-anther distance and betalain content in petals were notably high: 79.77% and 61.87% for ‘HHQL’ × ‘HL’ and 77.02% and 51.12% for ‘HL’ × ‘HHQL’, respectively. The wide variation indicates substantial genetic segregation in these hybrid populations, supporting the potential for trait selection, indicative of a broad genetic diversity that may facilitate trait selection. The significant genetic transmission of stigma-to-anther distance and betalain content further indicates that these traits are primarily influenced by genetic factors, with minimal environmental impact.

Additionally, both cross combinations demonstrated positive hybrid advantage rates for perianth width and stigma-to-anther distance, revealing substantial hybrid vigor. Particularly for stigma-to-anther distance, the advantage rates reached 108.06% for ‘HHQL’ × ‘HL’ and 60.20% for ‘HL’ × ‘HHQL’. These findings highlight the potential for hybrid breeding to enhance floral characteristics such as size and pigmentation, supporting the selection of favorable genetic traits for improved pitaya varieties.

#### 3.2.4. Genetic Analyses of Main Genes and Multigene of Flower Traits

A comprehensive joint analysis was performed on seven flower traits, i.e., flower length, perianth width, calyx tube width, stigma-to-anther distance, betalain content in petals, and the number of petals and stigma lobes within the F1 progenies. This analysis employed a multi-gene mixed inheritance model to evaluate both major and polygenic effects on these quantitative traits. The Akaike Information Criterion (AIC) was used to balance model complexity with the fit to the observed data, providing an objective means to identify the most suitable model. Based on the Iterative Estimation of Conditional Mode (IECM) algorithm, eleven potential models were generated and evaluated. Models with the lowest AIC value, along with those closest to this minimum, were selected as optimal or alternative models for further analysis (Supplementary Table S7). This approach enables a refined understanding of the genetic architecture underlying key flower traits in pitaya hybrids.

Flower length in the F1 progenies of the ‘HHQL’ × ‘HL’ was analyzed to identify optimal genetic models by using the AIC minimum criterion. Models with the lowest AIC values specifically, the 0MG, 2MG-EA, and 2MG-AD models were chosen for further validation through suitability tests, including the homogeneity test (U12, U22, U32), the Smirnov test (nW2), and the Kolmogorov test (Dn), as shown in Supplementary Table S8. The 2MG-EA model, which assumes two pairs of equal additive major genes, was found to be the best fit, making it the optimal model for flower length in the ‘HHQL’ × ‘HL’ progenies.

Notably, the optimal genetic models differed for various traits across the reciprocal cross combinations. In the ‘HHQL’ × ‘HL’ hybrids, flower length and the number of stigma lobes were best represented by the 2MG-EA model, while the stigma-to-anther distance was better captured by the 2MG-AD model, which involves additive-dominant gene interactions. Furthermore, the ‘HHQL’ × ‘HL’ cross showed a higher number of stigma lobes than the reciprocal ‘HL’ × ‘HHQL’ cross, suggesting a genetic tendency influenced by the direction of the cross. For traits such as perianth width, calyx tube width, and petal number, the 2MG-EA model provided the most accurate fit, indicating control by two pairs of equal additive genes. In contrast, petal color was optimally described by the 2MG-AD model, implying that this trait is regulated by two pairs of additive-dominant genes. This detailed genetic modeling of floral traits in pitaya hybrids offers critical insights for selective breeding, enabling the development of varieties with targeted flower characteristics.

The genetic parameters of the optimal genetic model are shown in Table 2. For perianth width, the heredity was controlled by two pairs of equal additive major genes (2MG-EA), and the heritability of major genes was 69.55% and 87.59% in ‘HHQL’ × ‘HL’ and ‘HL’ × ‘HHQL’ cross combinations, respectively. Similarly, flower length and calyx tube width were consistent with the model of two pairs of equal additive major genes; the heritability of major genes was 16.18% and 45.91%, respectively. In the ‘HL’ × ‘HHQL’ cross combination, the additive effects of the first pair of genes were 1.7029% and 0.181%, respectively, while the heritability of major genes was 99.0503% and 62.2776%, respectively. The distance between stigma and anther, and betalain content in petals conformed to the model of two pairs of additive-dominant major genes. The two pairs of additive major genes were positive, while the dominant ones were negative.

### 3.3. Fruit Traits

#### 3.3.1. Fruit Weight, TSS Content, Peel and Pulp Color

In F1 progenies of ‘HHQL’ × ‘HL’ and ‘HL’ × ‘HHQL’ cross combinations, rich genetic variation was detected in various traits. The maximum fruit weights in ‘HHQL’ × ‘HL’ and ‘HL’ × ‘HHQL’ cross combinations were 192.16 g and 182.62 g, respectively, while the minimum weights were 26.08 g and 31.29 g. Fruit weights were predominantly distributed between 50–130 g, representing 73.0% of the F1 progenies. The average fruit weights of F1 progenies were lower than those of the average parents’ values, with coefficients of variation of 38.27% and 40.04%, respectively. The F1 progenies exhibit wide separation in fruit weights and show a decreasing trend in fruit size. The heritability was 72.02%, indicating that the variation was mainly from genetic effects with minimal environmental influence. In the reciprocal cross populations, the highest TSS content was 22.15% and 22.17% for ‘HHQL’ × ‘HL’ and ‘HL’ × ‘HHQL’ cross combinations, respectively, compared with the lowest values of 11.30% and 9.50%. The average TSS content was lower than that of the parents, indicating a tendency towards inferior quality in terms of TSS content.

**Figure 4:**
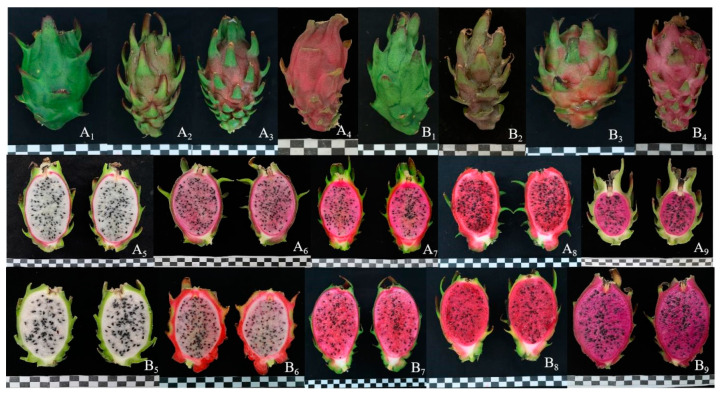
Peel and pulp color of F1 progenies from ‘HHQL’ × ‘HL’ (**A**) and ‘HHQL’ × ‘HL’ (**B**) 1, green peel; 2, green–red peel; 3, red–green peel; 4, red peel; 5, white; 6, light pink; 7, pink; 8, red; 9, purple-red.

The fruit peel exhibited four different colors, i.e., green, green–red, red–green, and red (Figure 4(A_1_–A_4_,B_1_–B_4_)), accounting for 69.3%, 18.7%, 6.0%, and 6.0% in F1 progenies of ‘HHQL’ × ‘HL’ cross combination compared with 66.0%, 20.0%, 6.0%, and 8.0% for ‘HL’ × ‘HHQL’, respectively. The majority of peel color was green, indicating a tendency towards the inheritance of ‘HHQL’ pitaya. The pulp colors were white, light pink, pink, red, and purple–red (Figure 4(A_5_–A_9_,B_5_–B_9_)). The pulp color heritability of F1 progenies from ‘HHQL’ × ‘HL’ and ‘HHQL’ × ‘HL’ cross combinations was 56.17% and 79.64%, respectively, mainly influenced by genetic effects.

Analysis of pulp color in both ‘HHQL’ × ‘HL’ and ‘HL’ × ‘HHQL’ cross populations revealed a predominant trend towards white pulp. In the ‘HHQL’ × ‘HL’ cross, 67.3% of the F1 progeny exhibited white pulp, while in the reciprocal ‘HL’ × ‘HHQL’ cross, 53.0% showed this trait. This consistency across both crosses, with more than half of the F1 plants displaying white pulp, underscores a strong genetic inclination towards the white-pulp trait inherited from the ‘HHQL’ parent. These findings suggest that white pulp is likely a dominant characteristic in these hybrid combinations, providing valuable insights for future breeding aimed at enhancing pulp color traits in pitaya varieties.

#### 3.3.2. Distributions of Main Fruit Traits

**Figure 5:**
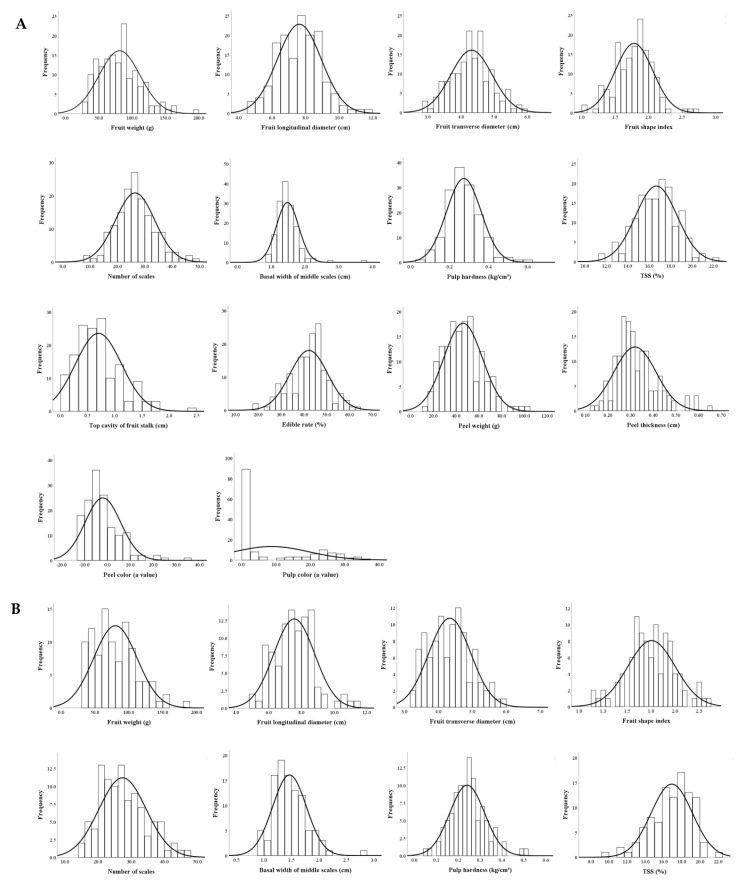

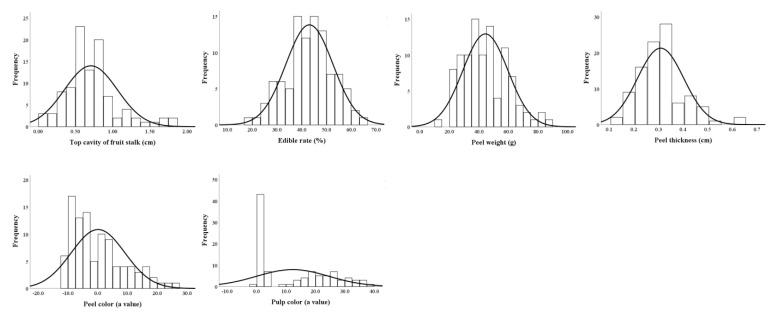
Frequency distribution of fruit main traits from ‘HHQL’ × ‘HL’ (**A**) and ‘HL’ × ‘HHQL’ (**B**) cross combinations.

A comprehensive analysis was conducted on fourteen key fruit traits in F1 progenies from the ‘HHQL’ × ‘HL’ and ‘HL’ × ‘HHQL’ cross combinations. These traits included fruit weight, longitudinal and transverse diameters, shape index, scale count, basal width of middle scales, flesh hardness, TSS content, top cavity of the fruit stalk, edible rate, peel weight, and thickness, as well as peel and pulp color. All traits displayed either normal or skewed normal distribution patterns (Figure 5), indicating that they are quantitative traits likely controlled by multiple genes. This distribution suggests that these fruit characteristics are influenced by a combination of genetic factors, offering valuable insights for breeding programs aimed at optimizing fruit quality and yield in pitaya.

#### 3.3.3. Genetic Analyses of Fruit Traits

The 14 fruit traits exhibited a certain degree of variation, with all traits showing high heritability, suggesting that the variation in these 14 fruit traits was mainly influenced by genetic effects (Table 3). The significant variation coefficients in the fruit top cavity, fruit peel, and pulp color indicated the extensive segregation in the offspring. The indices for fruit shape index, pulp hardness, and the dominance rate of the fruit top cavity were all positive, demonstrating a certain degree of hybrid vigor. The mean values of fruit weight, fruit transverse diameter, number of scales, basal width of middle scales, TSS content, edible rate, peel weight, peel thickness, peel, and flesh color in the F1 hybrid population were all lower than the average value of parents, showing a trend of inferior variation in fruit size and TSS content.

#### 3.3.4. Genetic Analyses of Main Genes and Multigene of Fruit Traits

Genetic analysis was performed on 14 fruit main traits of F1 progenies from ‘HHQL’ × ‘HL’ and ‘HL’ × ‘HHQL’ cross combinations using major genes plus polygenes. The model with the smallest or relatively small AIC value (Supplementary Tables S9 and S10) was selected as the alternative model. Suitability tests were conducted on the alternative models using U_1_^2^, U_2_^2^, U_3_^2^, nW^2^, and Dn (Supplementary Tables S11 and S12), resulting in the identification of the most suitable genetic model for each trait.

The genetic model of fruit shape index, pulp hardness, TSS content, peel weight, peel color, and pulp color from the ‘HHQL’ × ‘HL’ and ‘HL’ × ‘HHQL’ cross combinations were consistent, while the optimal models for other eight traits were different. Due to the larger number of offspring in the ‘HHQL’ × ‘HL’ cross combination, the results of the ‘HHQL’ × ‘HL’ cross combination were considered. The most suitable model for fruit weight, shape index, basal width of middle scales, and peel thickness was the 2MG-A, controlled by two pairs of additive major genes. The best-fit model for TSS content fruit longitudinal and transverse diameter was the 2MG-EA model, which was controlled by two pairs of equal additive major genes. 1MG-A was the best-fit model for a number of scales and flesh hardness. The most suitable model for the top cavity of fruit, edible rate, peel color, and peel weight was 2MG-AD, controlled by two pairs of additive–dominant major genes. The genetic model suitable for pulp color was 1MG-AD.

In the reciprocal cross populations, additive effects were positive and exceeded dominant effects for most fruit traits, with the exception of the first pair of major genes controlling fruit weight in the ‘HHQL’ × ‘HL’ population. This suggests that additive effects primarily regulate these fruit traits, promoting a consistent positive influence across generations. Notably, the genetic coefficients for sepal number, TSS content, pulp hardness, peel weight, and pulp color were positive in both ‘HHQL’ × ‘HL’ and ‘HL’ × ‘HHQL’ crosses, reflecting stable inheritance patterns. The genetic rates of the main genes responsible for TSS content, edible rate, and pulp color were comparatively high (Table 4), indicating that these traits are largely governed by genetic factors and exhibit low environmental sensitivity. This genetic stability offers promising insights for breeding programs focused on enhancing these specific fruit quality traits in pitaya.

### 3.4. Stem Traits

#### 3.4.1. Distributions of Stem Traits

**Figure 6:**
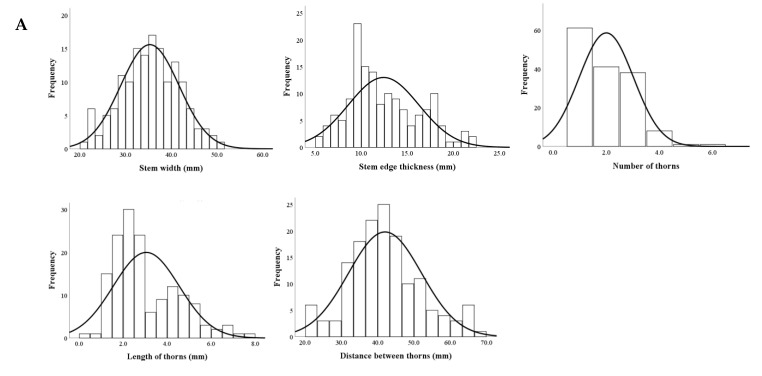

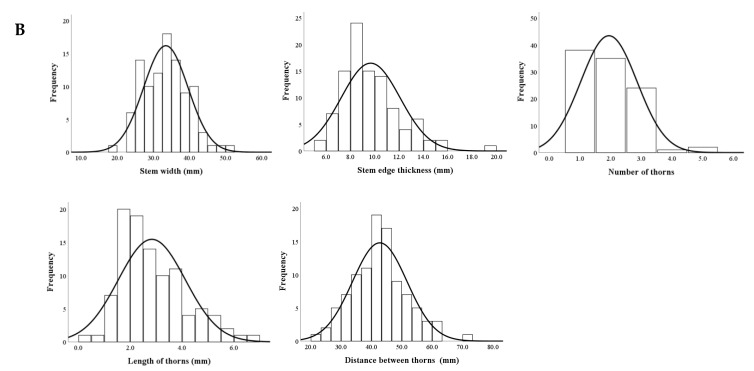
Frequency distributions of stem traits from ‘HHQL’ × ‘HL’ (**A**) and ‘HL’ × ‘HHQL’ (**B**) cross combinations.

Five key stem traits including stem width, edge thickness, thorn number, thorn length, and distance between thorns were analyzed in the F1 progenies of the ‘HHQL’ × ‘HL’ and ‘HL’ × ‘HHQL’ cross combinations. Each of these traits exhibited a normal distribution, suggesting that they are quantitative traits likely influenced by multiple genes. This distribution pattern highlights the potential for controlled genetic selection in future breeding efforts to optimize these stem characteristics for improving growth and resilience in pitaya (Figure 6).

#### 3.4.2. Genetic Analyses of Stem Traits

The average values of stem width and stem edge thickness of F1 progenies from ‘HHQL’ × ‘HL’ cross combination were 35.37 mm and 12.26 mm compared with 33.62 mm and 9.78 mm for ‘HL’ × ‘HHQL’ cross combination, respectively, which was higher than that of their parents of ‘HHQL’ and ‘HL’ pitayas.

The coefficients of variation for the number and length of thorns were higher than 40.00%; the heritability of five traits surpasses 90.00% (Table 5), indicating the variation in these traits was mainly influenced by genetic effects. The dominance rates of stem width, stem edge thickness, thorn number, and length were positive for both cross combinations; especially the dominance rates of stem edge thickness were 56.83% and 25.12%, respectively, indicating that stem exhibited a wider and more robust hereditary trend.

#### 3.4.3. Some Pseudo Qualitative Traits and the Infection Status of Canker Disease

**Figure 7:**
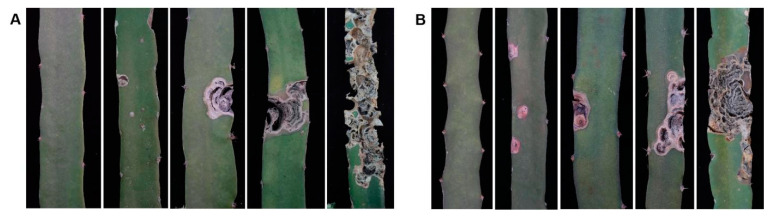
Infection degree of canker disease stems from ‘HHQL’ × ‘HL’ (**A**) and ‘HL’ × ‘HHQL’ (**B**) cross combinations.

The classification distribution of the degree of infection canker disease in F1 progenies from ‘HHQL’ × ‘HL’ and ‘HL’ × ‘HHQL’ cross combinations are shown in Table 6. There was a higher proportion of individual plants with a degree of infection in canker disease (level 2), while the severe infection of canker disease was a smaller proportion (Figure 7). The percentage of individuals with lower disease infection (levels 0 and 1) is 43.4% and 38.0%, respectively.

#### 3.4.4. Genetic Analyses of Main Genes and Multigene of Stem Traits

A mixed genetic analysis was conducted on five quantitative traits of stems from F1 progenies from ‘HHQL’ × ‘HL’ and ‘HL’ × ‘HHQL’ cross combinations (Supplementary Tables S13 and S14). The model controlling the five traits showed slight differences, thus the larger F1 progenies of the ‘HHQL’ × ‘HL’ cross combination were considered.

The model suitable for stem width and number of thorns was 2MG-EA, controlled by two pairs of additive major genes. The optimal model for stem thickness and length of thorns was 2MG-AD, controlled by two pairs of additive–dominant major genes. The best model for the distance between thorns was 1MG-AD, controlled by one pair of additive–dominant major genes (Supplementary Table S15). The five quantitative traits of stems in the ‘HHQL’ × ‘HL’ and ‘HL’ × ‘HHQL’ cross combinations were mainly influenced by positive additive effects. The main gene heritability was higher for the distance between thorns (Table 7).

### 3.5. Correlation Analysis of Flower and Fruit Traits

**Figure 8:**
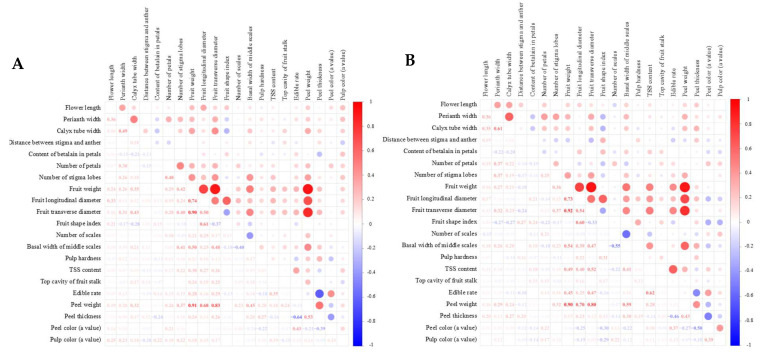
Correlation analysis of flower and fruit traits from ‘HHQL’ × ‘HL’ (**A**) and ‘HL’ × ‘HHQL’ (**B**) cross combinations. Circle sizes and color shade stand for degree of correlation. The lager circle sizes and the darker color mean the more significant degree of correlation.

Correlation analysis of flower and fruit traits revealed notable relationships between flower size and fruit size. Flower size showed a strong positive correlation with single fruit weight, indicating that larger flowers may contribute to increased fruit mass. Additionally, perianth width and calyx tube width were positively correlated with the fruit’s horizontal diameter. Single fruit weight also demonstrated a strong positive correlation with both the longitudinal and horizontal diameters of the fruit, as well as with peel weight. Conversely, a high negative correlation was observed between the number of scales and the basal width of the middle scales. Fruit peel thickness showed a strong positive correlation with fruit skin weight while displaying a high negative correlation with edible rate, suggesting that thicker peels may reduce the proportion of edible fruit content (Figure 8).

### 3.6. Screening of New Germplasm

Since both ‘HHQL’ and ‘HL’ pitaya are self-incompatible with small fruits, no individual plant with good fruit characteristics was selected from the F1 progenies. However, ‘HHQL’ pitaya is notable for its early flowering, typically blooming in mid-April. In the F1 progenies from both ‘HHQL’ × ‘HL’ and ‘HL’ × ‘HHQL’ cross combinations, some plants demonstrated even earlier flowering, beginning as early as late March. Specifically, in the ‘HHQL’ × ‘HL’ cross, two plants with very short thorns, five early flowering plants, and two with reduced symptoms of canker disease were identified (Table 8). Three of these plants were further selected for their combined traits of early flowering and disease resilience. Similarly, in the ‘HL’ × ‘HHQL’ cross progenies, three plants with very short thorns, three thornless plants, two early flowering plants, and two plants exhibiting both early flowering and lower canker disease severity were identified. These selections highlight valuable traits for future breeding, particularly for early flowering, thorn reduction, and disease resistance. 

## 4. Discussion

### 4.1. The Genetic Effect of Flower Color

Petals are the most visually appealing and ornamental structures of flowers. In fruit trees, variations in petal color and shape play a critical role in attracting pollinators that directly affect the pollination process, fertilization, and ultimately fruit yield [35]. Commercially grown pitaya varieties typically have white flowers, which may limit their attractiveness to pollinators, potentially impacting the pollination process. In this study, we developed F1 progenies using the ‘HHQL’ pitaya as a parental line to introduce and explore variations in flower color within the F1 generation. This approach aims to support breeding efforts focused on enhancing ornamental value and improving pollination efficiency, ultimately facilitating the development of highly ornamental pitaya varieties with diverse and vibrant flower colors.

Betalain is responsible for petal coloration in pitaya, and more betalain results in darker and a more reddish color [33]. F1 generation of red-flowered and white-flowered individuals exhibited white petal color in more than half of the entire population, suggesting the white color genes could be dominant in offspring. However, the red color of the petals varied among individuals showing a wide range of genetic variations. Similar findings have been reported in kiwifruit, cherry [29], and lemon [36]. Previous studies showed the occurrence of darker petal colors in the ‘DH’×‘HHQL’ and ‘HHQL’ × ‘DH’ offspring populations suggesting the involvement of maternal inheritance influencing petal pigmentation. The absence of darker petals in the ‘HL’ × ‘HHQL’ cross supports the significance of the maternal parent in determining petal color intensity. The predominance of white flowers in the ‘HHQL’ × ‘DH’ F1 generation further underscores the dominant nature of the white petal trait from ‘DH’ [33]. Zhou et al. [37] reported similar findings in an ornamental plant *Hedychium coronarium.* Therefore, darker color petals of parental flowers induce a higher probability of darker petal color in their offspring after hybridization as reported by Zhu et al. [38] in the analysis of flower traits inheritance in *Phalaenopsis*. Moreover, the use of red color petal varieties could be an effective and beneficial tool to increase the hybridization and breeding of pitaya with specialized red petal colors.

### 4.2. The Genetic Effect of Flower Types

Different plant species have variations in flower size and number and sizes of petals that directly influence overall flower appearance. Two pairs of additive–dominant–epistatic major genes controlled floral traits in roses [39], peonies [40], and chrysanthemums [41]. In this study, the genetic control of petal traits in pitaya showed a unique mechanism influenced by two pairs of major genes. Flower size, typically represented by flower length, perianth width, and calyx tube width, was notably smaller in the hybrid offspring, with floral traits trending towards smaller dimensions. Similar findings have been reported in hybrid progenies of jujube and sour jujube, where floral traits exhibited comparable genetic tendencies [30]. Pitaya floral traits have seemed to be controlled by the 2MG-EA model, regulated by two pairs of additive-major genes as reported by previous studies suggesting positive or negative dominant effects of major genes [42]. These additive effects, passed on to subsequent generations, provide a valuable basis for achieving targeted breeding outcomes in controlled quantitative traits.

### 4.3. The Genetic Effect of Stigmas

Fruit growth, development, and size are significantly affected by floral traits [43]. The quantity and quality of pollen are important factors affecting pollination and fertilization of fruit trees [44]. Distance between stigma and pollens and their sizes are crucial for pollination and fertilization. Lower or the same height of stigma to anthers increases the proportion of self-pollination without the need for wind, insects, or other ways. The stronger degree of stigma opening and larger contact area with pollen during pollination is more conducive to pollination [45]. Therefore, the traits of stigma, processes of pollination and fertilization, as well as the traits of the fruit were found to be closely related to each other. Therefore, the present study depicted that F_1_ progenies of ‘HHQL’ × ‘HL’ and ‘HHQL’ × ‘HL’ cross combinations had higher stigma numbers and less distance between stigma and anthers, indicating a stronger genetic influence than environmental. Thus, our findings reaffirm the results presented by Zhang et al. [33].

### 4.4. Genetic Analysis of Fruit Traits

Genetic tendency analysis of the ‘HHQL’ × ‘HL’ and ‘HL’ × ‘HHQL’ cross combinations revealed that the offspring population exhibited smaller fruit sizes compared to the maternal parent. This trend aligns with findings in previous studies, which have similarly observed a genetic tendency towards reduced fruit size in hybrid offspring across various fruit tree species. Such tendencies highlight the influence of genetic factors in determining fruit size and underscore the potential need for selective breeding strategies to achieve desired fruit dimensions in pitaya [46,47]. In this study, the average fruit weight in the progeny with ‘HHQL’ (80.88 g) as the maternal parent was slightly higher and had a wider distribution range than the back cross population (79.99 g). A similar pattern was observed in the ‘HHQL’ × ‘DH’ hybrid population, suggesting maternal inheritance influences fruit size, affected by the small-fruited parent. This phenomenon of maternal inheritance in fruit size is also reported in cranberries [48] and intergeneric hybrids of lychee and longan.

The slight difference in average fruit weight between the two hybrid offspring groups likely stems from the smaller size of the parent fruits. Selecting larger-fruited parent varieties would be more effective for breeding larger fruits. Future research should prioritize varieties with notable differences in fruit weight for reciprocal crosses and expand offspring populations to further explore the role of maternal inheritance in determining fruit size in pitaya. Additionally, the reciprocal hybrid populations exhibited a higher and more variable average fruit shape index compared to the parental average, indicating a genetic tendency toward the development of more oval-shaped fruits. These findings are in line with the outcomes of Wu et al. [49] on apricots and Wei et al. [50] on pitaya.

### 4.5. The Appearance Quality of the Fruit

The offspring groups displayed higher mean values and a high coefficient of variation for the top cavity of the fruit compared to mid-parental values, indicating robust genetic transmission of this trait. This trend suggests an increase in top cavity length, which may contribute to a reduced likelihood of fruit cracking, thereby potentially enhancing fruit durability and quality [51]. In this study, crossing green-peel and red-peel parents produced offspring with mixed green and red peels. Reciprocal crosses resulted in more green-peel fruits, indicating that the green peel color is influenced by ‘HHQL’ and dominant over red. In the ‘HHQL’ × ‘DH’ hybrid population, fruit peel color tended towards green. Conversely, in the ‘DH’ × ‘HHQL’ population, more red-peel individuals appeared, suggesting a stronger maternal influence on peel color. This may be due to differences in red-peel varieties and smaller population size as ‘HL’ red-peel maternal parents. Moreover, crossing yellow-peel dragon fruit with two red-peel varieties produced no yellow-peel offspring which indicated red peel color was dominant over yellow. Usually, pitaya peels are red, green, and yellow but the genetic patterns behind color are not fully understood. Therefore, further research with various cross-combinations and larger offspring populations is needed to clarify these genetic patterns.

### 4.6. The Genetic Effect of TSS Content, Flesh Hardness and Color

The flesh firmness of ‘HHQL’ was higher than ‘HL’. The progeny from reciprocal crosses showed large variability and higher average values of flesh firmness than mid-parent values with heritability over 100%, which represented strong heterosis. These findings are consistent with Gala apples and Zhao [52] on pears flesh firmness of hybrid offspring. Higher fruit firmness within certain limits enhances disease resistance, storage, and transportation capabilities and reduces cracking [53]. Both flesh firmness and top cavity size directly affect pitaya cracking and should be prioritized in breeding programs.

The TSS content is crucial for fruit quality and often deteriorates in various fruit trees. In the present study, the average TSS content values of ‘HHQL’ × ‘HL’ and ‘HL’ × ‘HHQL’ populations were lower than mid-parent values but slightly higher than low-parent values, with heritability over 90%. Genetic transmission showed minimal environmental influence and small variation. Similar findings have been reported by Liu et al. (2020) on apricot’s reciprocal cross populations and Lei et al. [54] on blueberry TTS content of reciprocal progeny. This study identified 10 high-parent single plants in one group and 12 in another, with an average TSS content of 22.25%. This suggests the potential possibility of breeding individual plants with high TSS content.

In the present study, hybrid offsprings showed five fruit flesh colors: white, light pink, pink, purple-red, and red. More plants in both reciprocal crosses have white flesh, indicating a trend towards whitening in offspring. The cross combinations of ‘HHQL’ × ‘DH’, and ‘DH’ × ‘HHQL’ indicated the controlling white flesh color gene was dominant [33]. Thus, ‘HHQL’ significantly influences fruit peel and flesh colors in both reciprocal cross populations. Our study provides a reference for the selection of pitaya hybrid parents in the future and demonstrates that using a mixed genetic model for single segregation F_1_ generation analysis of dragon fruit traits is achievable. 

### 4.7. Genetic Analyses of Stem Traits

The results of the current study demonstrated that the hybrid offspring of ‘HHQL’ and ‘HL’ pitayas exhibited wider and thicker stems, traits inherited from both parents that contribute to enhanced robustness and potential disease resistance. These structural traits not only support overall plant resilience but also create a more nutrient-abundant environment, which promotes improved flowering and fruiting potential. Additionally, thorn traits in the hybrids showed decreased variability in both spacing and length, indicating intermediate heredity, which could simplify maintenance and harvesting while preserving some level of natural protection. Moreover, other stem traits resembled those of ‘HHQL’ plants. Previous studies found that the parental variety ‘HL’ is resistant while ‘HHQL’ is susceptible to the canker disease. The hybrid offspring show variability in disease susceptibility and thorn length, with some resistant to canker disease and having short thorns. Breeding for fewer and shorter thorns is crucial for efficient field work with more production. Comprehensive analysis reveals that a hybrid offspring population combines the advantages and characteristics of the parents, resulting in the possibility of selecting individual plants with strong stems and short thorns that are easy to manage in agricultural operations along with economic benefits.

## 5. Conclusions

The current investigation provides a comprehensive analysis of the genetic variation, segregation patterns, and inheritance models of flower, fruit, and stem traits in F1 progenies from the ‘HHQL’ × ‘HL’ and ‘HL’ × ‘HHQL’ cross combinations. The results revealed that seven quantitative flower traits exhibited segregation patterns favoring the ‘HL’ parent, with a notable trend towards smaller flower sizes. Traits such as stigma-to-anther distance and petal color showed high variation coefficients, exceeding 50%, which opens new opportunities for breeding pitaya plants with self-pollination potential and diverse flower colors. Additionally, over half of the F1 progenies showed a combination of green peel and white flesh, while fruit traits generally trended towards smaller size and lower total soluble solids (TSS) content, a critical factor for taste and marketability. The F1 progenies also displayed significant genetic variability in stem traits, particularly in five key stem characteristics, enabling the potential selection of plants with stronger stems and shorter thorns, both desirable for cultivation. Mixed genetic analyses highlighted the significant role of major gene additive effects in regulating these quantitative traits across flowers, fruits, and stems, underscoring the value of targeted genetic selection.

These findings underscore the substantial genetic diversity present in pitaya and the influence of major gene effects, offering valuable guidance for parent selection and the development of tailored cross-breeding programs. Future breeding efforts can leverage these insights to enhance key ornamental and agricultural traits in pitaya, ultimately contributing to the production of varieties with improved fruit quality, self-pollination capabilities, and resilience.

## Figures and Tables

**Table 1:** Genetic analyses of flower traits from ‘HHQL’ and ‘HL’ cross combinations.

Flower Traits	Cross Combinations	Parents		F_1_ Progenies				
		HHQL	HL	MP	F ± S	*CV* (%)	*Ta* (%)	*Ha* (%)
Flower length (cm)	Q × H	20.86	26.53	23.70	22.68 ± 1.85	8.17	95.71	−4.29
	H × Q			23.70	21.64 ± 1.9	8.80	91.32	−8.68
Perianth width (cm)	Q × H	3.79	4.90	4.35	4.73 ± 0.44	9.33	108.90	8.90
	H × Q			4.35	4.61 ± 0.5	10.83	106.14	6.14
Calyx tube width (cm)	Q × H	2.25	2.36	2.30	2.3 ± 0.24	10.42	99.94	−0.06
	H × Q			2.30	2.19 ± 0.22	10.15	94.90	−5.10
Distance between stigma and anther (cm)	Q × H	0.94	0.00	0.47	0.98 ± 0.78	79.77	208.06	108.06
	H × Q			0.47	0.75 ± 0.58	77.02	160.20	60.20
Content of betalain in petals (mg/100 g FW)	Q × H	5.77	1.00	3.38	2.3 ± 1.42	61.87	67.86	−32.14
	H × Q			3.38	2.11 ± 1.08	51.12	62.33	−37.67
No. of petals	Q × H	13.71	18.50	16.11	15.23 ± 2.03	13.33	94.58	−5.42
	H × Q			16.11	15.61 ± 1.85	11.84	96.89	−3.11
No. of stigma lobes	Q × H	19.57	16.75	18.16	16.91 ± 2.08	12.30	93.09	−6.91
	H × Q			18.16	17.22 ± 1.87	10.85	94.84	−5.16

Note: MP, the mid-parent value; F, average value of the F1 generation; S, standard deviation; *CV*, coefficient of variation; *Ta*, heritability; *Ha*, dominance ratio.

**Table 2:** Estimation of genetic parameters for different flower main traits at their optimal genetic models.

Traits	Cross Combination	Model	*m*	*da*	*db*	*ha*	*hb*	*i*	*jab*	*jba*	*l*	*pσ* ^2^ *mg*	*Hmg*^2^ (%)
Flower length	Q × H	2MG-EA	22.6873	1.5358								0.5471	16.1798
	H × Q	2MG-AD	21.0861	1.7029	0.4173	−0.79	2.1996					3.429	99.0503
Perianth width	Q × H	2MG-EA	4.7558	0.3138								0.1382	69.5537
	H × Q	2MG-EA	4.6074	0.3262								0.2187	87.5941
Calyx tube width	Q × H	2MG-EA	2.3024	0.2027								0.026	45.9066
	H × Q	2MG-EA	2.193	0.181								0.0278	62.2776
Distance between stigma and anther	Q × H	2MG-AD	1.149	0.8179	0.3312	−0.2547	−0.0661					0	0
	H × Q	2MG-EA	0.779	0.3895								0.0996	29.6612
Petal color	Q × H	2MG-AD	2.8421	1.6813	0.1343	−0.8732	0.035					0	0
	H × Q	2MG-AD	2.4486	1.2516	0.2333	−0.5821	−0.0839					0	0
No. of petals	Q × H	2MG-EA	15.2361	0.8835								0	0
	H × Q	2MG-EA	15.6832	1.5893								0	0
No. of stigma lobes	Q × H	2MG-EA	16.9195	1.1422								0	0
	H × Q	2MG-AD	17.7536	1.8213	1.0014	−0.9967	0.0076					3.3319	95.331

Note: *m*, group mean square; *da*, additive effect of the first pair of major genes; *db*, additive effect of the second pair of major genes; *ha*, dominance effect of the first pair of major genes; *hb*, dominance effect of the second pair of major genes; *i*, additive–additive effect; *jab*, additive–dominance effect; *jba*, dominance–additive effect; *l*, dominance–dominance effect; *pσ*^2^*mg*, major gene variance; *Hmg*^2^, major gene heritability.

**Table 3:** Genetic analyses of fruit main traits of ‘HHQL’ × ‘HL’ and ‘HL’ × ‘HHQL’ cross combinations.

Fruit Traits	Cross Combinations	Parents		F_1_				
		‘HHQL’	‘HL’	MP	F ± S	*CV* (%)	*Ta* (%)	*Ha* (%)
Fruit weight (%)	Q × H	155.69	68.92	112.30	80.88 ± 30.95	38.27	72.02	−27.98
	H × Q				79.99 ± 32.03	40.04	71.23	−28.77
Fruit longitudinal diameter (cm)	Q × H	9.27	5.90	7.59	7.62 ± 1.31	17.22	100.44	0.44
	H × Q				7.55 ± 1.25	16.61	99.56	−0.44
Fruit transverse diameter (cm)	Q × H	5.25	4.65	4.95	4.32 ± 0.62	14.39	87.14	−12.86
	H × Q				4.33 ± 0.62	14.26	87.52	−12.48
Fruit shape index	Q × H	1.76	1.27	1.52	1.78 ± 0.28	15.76	117.4	17.4
	H × Q				1.75 ± 0.25	14.11	115.62	15.62
Number of scales	Q × H	22.76	36.18	29.47	26.45 ± 7.19	27.17	89.75	−10.25
	H × Q				27.39 ± 7.12	25.99	92.95	−7.05
Basal width of middle scales (cm)	Q × H	2.53	0.53	1.53	1.48 ± 0.33	22.16	96.69	−3.31
	H × Q				1.46 ± 0.31	21.01	95.51	−4.49
Pulp hardness (kg/cm^3^)	Q × H	0.24	0.12	0.18	0.27 ± 0.09	32.51	149.75	49.75
	H × Q				0.24 ± 0.08	33.08	132.26	32.26
TSS (%)	Q × H	19.39	16.32	17.85	16.64 ± 1.96	11.75	93.18	−6.82
	H × Q				16.89 ± 2.26	13.4	94.61	−5.39
Top cavity of fruit (cm)	Q × H	0.86	0.43	0.64	0.7 ± 0.42	60.77	108.67	8.67
	H × Q				0.71 ± 0.36	50.39	110.12	10.12
Edible rate (%)	Q × H	43.87	57.71	50.79	42.18 ± 8.32	19.72	83.05	−16.95
	H × Q				43.07 ± 9.52	22.1	84.8	−15.2
Peel weight (g)	Q × H	87.59	29.08	58.34	46.05 ± 16.95	36.8	78.94	−21.06
	H × Q				43.07 ± 9.52	22.1	84.8	−15.2
Peel thickness (cm)	Q × H	0.41	0.23	0.32	0.32 ± 0.09	29.23	99.41	−0.59
	H × Q				0.31 ± 0.09	30.39	96.2	−3.8
Peel color (a value)	Q × H	−11.26	24.74	6.74	−2.02 ± 7.79	−385.32	−30	−130
	H × Q				0.08 ± 9.01	10,632.86	1.26	−98.74
Pulp color (a value)	Q × H	1.18	29.56	15.37	8.63 ± 10.98	127.21	56.17	−43.83
	H × Q				12.24 ± 12.34	100.83	79.64	−20.36

Note: MP, the mid-parent value; F, average value of the F1 generation; S, standard deviation; *CV*, coefficient of variation; *Ta*, heritability; *Ha*, dominance ratio.

**Table 4:** Estimation of genetic parameters for different fruit main traits at their optimal genetic models.

Fruit Traits	Cross Combinations	Model	*m*	*da*	*db*	*ha*	*hb*	*i*	*jab*	*jba*	*l*	*p*σ^2^*mg*	*Hmg*^2^ (%)
Fruit weight	Q × H	2MG-A	81.4138	−5.2296	32.4847							0	0
	H × Q	2MG-AD	89.3111	37.3628	13.7251	−7.7391	−8.0902					328.9568	32.0647
Fruit longitudinal diameter	Q × H	2MG-EA	7.6034	0.7411								0	0
	H × Q	2MG-AD	7.6645	0.8943	0.8661	0.0599	−0.1903					0	0
Fruit transverse diameter	Q × H	2MG-EA	4.3152	0.1616								0	0
	H × Q	2MG-AD	4.5945	0.5498	0.4488	0.0343	−0.6234					0.3536	92.5371
Fruit shape index	Q × H	2MG-A	1.7872	0.0728	0.2888							0	0
	H × Q	2MG-A	1.7485	−0.1325	0.0048							0	0
Number of scales	Q × H	1MG-A	26.4444	7.0627								3.4187	6.6203
	H × Q	2MG-EA	27.2588	5.8099								32.7702	64.6687
Basal width of middle scales	Q × H	2MG-A	1.5046	0.0771	0.2648							0	0
	H × Q	2MG-EA	1.4625	0.2609								0	0
Flesh hardness	Q × H	1MG-A	0.2724	0.0576								0	0
	H × Q	1MG-A	0.2405	0.0516								0	0
TSS content	Q × H	2MG-EA	16.6353	0.6598								2.7346	71.5213
	H × Q	2MG-EA	16.967	1.3149								4.9581	96.7798
Top cavity of fruit	Q × H	2MG-AD	0.6663	0.4386	0.2277	−0.1472	0.201					0	0
	H × Q	2MG-EA	0.7287	0.2484								0	0
Edible rate	Q × H	2MG-AD	0.3497	0.0592	0.0186	0.0968	0.0482					0.0069	99.5973
	H × Q	2MG-EA	0.4336	0.0685								0.0086	95.0738
Peel weight	Q × H	2MG-AD	50.1727	12.7099	16.1696	0.4114	−8.9628					11.5839	4.0332
	H × Q	2MG-AD	50.6289	15.1547	9.873	−3.1703	−8.7904					176.0511	75.4654
Peel thickness	Q × H	2MG-A	0.315	0.0289	0.0849							0	0
	H × Q	2MG-AD	0.3331	0.069	0.0659	0.0118	−0.0618					0	0
Peel color	Q × H	2MG-AD	0.5249	6.1507	5.2031	−1.4405	−3.5765					0	0
	H × Q	2MG-AD	2.8634	10.5269	2.4549	−4.01	−0.6129					50.9704	62.8422
Flesh color	Q × H	1MG-AD	11.8026	10.9527		−10.0023						65.8128	54.552
	H × Q	1MG-AD	16.3114	14.8952		3.9782						136.5994	89.6459

Note: *m*, group mean square; *da*, additive effect of the first pair of major genes; *db*, additive effect of the second pair of major genes; *ha*, dominance effect of the first pair of major genes; *hb*, dominance effect of the second pair of major genes; *i*, additive–additive effect; *jab*, additive–dominance effect; *jba*, dominance–additive effect; *l*, dominance–dominance effect; *pσ*^2^*mg*, major gene variance; *Hmg*^2^, major gene heritability.

**Table 5:** Genetic analyses of stem traits from ‘HHQL’ × ‘HL’ and ‘HL’ × ‘HHQL’ cross combinations.

Traits	Cross Combinations	Parents		F_1_				
		‘HHQL’	‘HL’	MP	F ± S	*CV* (%)	*Ta* (%)	*Ha* (%)
Stem width	Q × H	39.91	23.65	31.78	35.37 ± 6.8	19.22	111.30	11.30
	H × Q				33.62 ± 6.14	18.27	105.78	5.78
Stem edge thickness	Q × H	4.85	10.79	7.82	12.26 ± 3.66	29.86	156.83	56.83
	H × Q				9.78 ± 2.83	28.88	125.12	25.12
No. of thorns	Q × H	1.68	1.80	1.74	2.0 ± 1.01	49.95	116.67	16.67
	H × Q				1.93 ± 0.9	46.94	110.75	10.75
Length of thorns	Q × H	4.16	1.50	2.83	2.98 ± 1.44	48.26	105.43	5.43
	H × Q				2.84 ± 1.26	44.29	100.51	0.51
Distance between thorns	Q × H	56.84	34.24	45.54	42.44 ± 10.06	23.71	93.18	−6.82
	H × Q				42.47 ± 8.55	20.12	93.25	−6.75

Note: MP, the mid-parent value; F, average value of the F_1_ generation; S, standard deviation; *CV*, coefficient of variation; *Ta*, heritability; *Ha*, dominance ratio.

**Table 6:** The segregation ratio of cankers disease in F1 progenies.

Infection Degree of Canker Disease	Number (Proportion%)	
0	23 (15.4)	10 (10.0)
1	42 (28.0)	28 (28.0)
2	62 (41.3)	33 (33.0)
3	17 (11.3)	20 (20.0)
4	6 (4.0)	9 (9.0)

**Table 7:** Estimation of genetic parameters for five stem traits at their optimal genetic models.

Traits	Cross Combinations	Model	*m*	*da*	*db*	*ha*	*hb*	*i*	*jab*	*jba*	*l*	*p*σ^2^*mg*	*Hmg*^2^(%)
Stem width	Q × H	2MG-EA	35.1176	6.5151								0	0
	H × Q	2MG-AD	37.8574	4.4315	4.0126	−1.1909	−6.6436					12.9563	34.3389
Stem edge thickness	Q × H	2MG-AD	13.9416	4.5009	−0.3552	−1.1979	−2.1556					9.5687	71.3481
	H × Q	2MG-EA	9.7159	1.7433								0	0
No. of thorns	Q × H	2MG-EA	2.4408	0.7204								0	0
	H × Q	2MG-A	2.1072	0.8603	−0.14							0	0
Length of thorns	Q × H	2MG-AD	3.4052	1.5201	0.5609	−0.882	0.0047					0.529	25.1501
	H × Q	1MG-AD	3.0022	1.1478		−0.4084						0	0
Distance between thorns	Q × H	1MG-AD	43.0431	11.1056		−1.1581						37.498	37.0371
	H × Q	2MG-AD	46.8981	6.7489	1.9961	−0.3493	−8.0521					22.0752	30.484

Note: *m*, group mean square; *da*, additive effect of the first pair of major genes; *db*, additive effect of the second pair of major genes; *ha*, dominance effect of the first pair of major genes; *hb*, dominance effect of the second pair of major genes; *i*, additive–additive effect; *jab*, additive–dominance effect; *jba*, dominance–additive effect; *l*, dominance–dominance effect; *pσ*^2^*mg*, major gene variance; *Hmg*^2^, major gene heritability.

**Table 8:** Screening of new germplasm.

Cross Combination Traits	Early Flowering *	Plant with Short Thorns	Plant Without Thorns	Weak of Infection Canker Disease *	Early Flowering with Weak Infection of Canker Disease
‘HHQL’ × ‘HL’	5	2	0	2	3
‘HL’ × ‘HHQL’	2	3	3	0	2

* Early flowering refers to individual plants observed for two years, showing the first batch of flowers opening from late March to early April. Weak infection canker disease indicates that the canker disease symptoms of the individual plants were at level 0 or 1.

## Data Availability

The original contributions presented in the study are included in the article/Supplementary Materials; further inquiries can be directed to the corresponding authors.

## Supplementary Materials

The following supporting information can be downloaded at: https://www.mdpi.com/article/10.3390/plants13243546/s1, Figure S1. The flower, fruit, and stem traits of ‘HHQL’ (A) and ‘HL’ (B) pitayas. Figure S2 Hybridization process. Figure S3 Identification results of F1 progenies of ‘HHQL’×‘HL’ and ‘HL’×‘HHQL’ cross combination. Table S1. Main flower and fruit traits of ‘HHQL’ and ‘HL’ pitayas. Table S2. Comparison of stem traits of ‘HHQL’ and ‘HL’ pitayas. Table S3. SCoT primers used in this study. Table S4. 17 pairs of SRAP molecular marker primer. Table S5. SRAP primers used in this study. Table S6. Disease index of pitaya canker disease. Table S7. The AIC values of flower main traits of F_1_ progenies from ‘HHQL’ × ‘HL’ and ‘HL’ × ‘HHQL’ cross combinations under different genetic models. Table S8. Optimal model for flower main traits of F1 progenies from ‘HHQL’ × ‘HL’ and ‘HL’ × ‘HHQL’ cross combinations using suitable test. Table S9. The AIC values of fruit main traits of ‘HHQL’ × ‘HL’ cross combinations under different genetic models. Table S10. The AIC values of fruit main traits of ‘HL’ × ‘HHQL’ cross combinations under different genetic models. Table S11. Optimal model for fruit main traits of F_1_ progenies from ‘HHQL’ × ‘HL’ cross combination using suitable test. Table S12. Optimal model for fruit main traits of F_1_ progenies from ‘HL’ × ‘HHQL’ cross combinations using suitable test. Table S13. The AIC values of stem traits from ‘HHQL’ × ‘HL’ and ‘HL’ × ‘HHQL’ cross combinations under different genetic models. Table S14. Optimal model of stem traits of F_1_ progenies from ‘HHQL’ × ‘HL’ cross combinations using suitable test. Table S15. Optimal model of stem traits of F_1_ progenies from ‘HL’ × ‘HHQL’ cross combinations using suitable test.
