# Supplementary material for: Genetic Analyses of Flower, Fruit, and Stem Traits of Intergeneric Hybrids Between ‘Honghuagqinglong’ and ‘Heilong’ Pitayas
Source: Plants (Basel). 2024 Dec 19;13(24):3546. doi: 10.3390/plants13243546 (PMC11680067; doi:10.3390/plants13243546)
Supplement: Supplementary file 1 [file plants-13-03546-s001.zip › Supplementary Table 14.pdf]

Supplementary Table S14. Optimal model of stem traits of F_1 progenies from 'HHQL' × 'HL' cross combinations using suitable test.

Traits	Model	AIC	U_1^2	U_2^2	U_3^2	nW^2	D^n
Stem width	2MG-AD	1367.768	0(0.9986)	0(0.999)	0.0001(0.9906)	0.0098(1.0001)	0.0222(1)
	2MG-EA	1364.14	0.0057(0.9397)	0.0038(0.951)	0.2904(0.5899)	0.022(0.9948)	0.0265(0.9987)
Stem edge thickness	2MG-AD	1077.813	0.0002(0.9885)	0(0.9994)	0.0028(0.9577)	0.0107(1)	0.0221(1)
	2MG-EA	1083.081	0.1061(0.7446)	0.1182(0.731)	0.0129(0.9094)	0.0373(0.9468)	0.042(0.8604)
No. of thorns	2MG-EA	-4814.303	4.1132(0.0425)	0.5462(0.4599)	23.9971(0.000001)	2.6814(0.0001)	0.305(0)
Length of thorns	2MG-AD	666.4185	0(0.9969)	0(0.9982)	0(0.9951)	0.0073(1.0014)	0.0182(1)
Distance between thorns	1MG-AD	1534.746	0(0.9975)	0(0.9979)	0.0005(0.9819)	0.0109(1)	0.0243(0.9997)
	2MG-AD	1535.218	0.0011(0.9735)	0.0048(0.9448)	0.0221(0.8819)	0.0113(1)	0.0265(0.9987)
	2MG-EA	1535.929	0.0059(0.9387)	0.0056(0.9404)	0(0.9991)	0.017(0.999)	0.0237(0.9998)
