# Supplementary material for: Genetic Analyses of Flower, Fruit, and Stem Traits of Intergeneric Hybrids Between ‘Honghuagqinglong’ and ‘Heilong’ Pitayas
Source: Plants (Basel). 2024 Dec 19;13(24):3546. doi: 10.3390/plants13243546 (PMC11680067; doi:10.3390/plants13243546)
Supplement: Supplementary file 1 [file plants-13-03546-s001.zip › Supplementary Table 15.pdf]

Supplementary Table S15. Optimal model of stem traits of F_1 progenies from 'HL' × 'HHQL' cross combinations using suitable test.

Traits	Model	AIC	U_1^2	U_2^2	U_3^2	nW^2	D^n
Stem width	2MG-AD	891.2013	0.0008(0.9768)	0.004(0.9495)	0.0197(0.8884)	0.0078(1.0008)	0.0252(1)
	2MG-EA	898.1331	0.0017(0.9667)	0.0001(0.9912)	0.0139(0.9063)	0.0203(0.9967)	0.0334(0.9977)
Stem edge thickness	2MG-AD	635.3495	0.0029(0.9572)	0.0006(0.9801)	0.0117(0.914)	0.0135(0.9998)	0.0349(0.9958)
	2MG-A	637.8454	0.0401(0.8413)	0.0369(0.8477)	0.0001(0.9941)	0.0241(0.9913)	0.036(0.9937)
	2MG-EA	634.2901	0.0231(0.8793)	0.0424(0.8369)	0.0552(0.8142)	0.0218(0.995)	0.0342(0.9969)
No. of thorns	2MG-A	-3471.365	3.9952(0.0456)	1.0557(0.3042)	13.1866(0.0003)	2.1169(0)	0.3172(0)
Length of thorns	1MG-AD	440.9772	0.0022(0.9626)	0.0005(0.9816)	0.008(0.9288)	0.0133(0.9999)	0.0335(0.9977)
	1MG-A	441.9137	0.0023(0.9617)	0.0004(0.9844)	0.0699(0.7915)	0.0192(0.9977)	0.0294(0.9997)
	2MG-A	441.9598	0.0022(0.9624)	0.0002(0.9893)	0.0165(0.8977)	0.0141(0.9998)	0.026(1)
Distance between thorns	1MG-A	983.0809	0.0002(0.989)	0.0009(0.9762)	0.0044(0.9473)	0.0207(0.9963)	0.0373(0.9906)
	2MG-AD	976.9417	0.0005(0.9825)	0.0004(0.9839)	0(0.9965)	0.0066(1.0034)	0.0217(1)
	2MG-A	982.9556	0.0677(0.7947)	0.0678(0.7945)	0.0012(0.9729)	0.0215(0.9953)	0.0436(0.956)
