# Supplementary material for: Genetic Analyses of Flower, Fruit, and Stem Traits of Intergeneric Hybrids Between ‘Honghuagqinglong’ and ‘Heilong’ Pitayas
Source: Plants (Basel). 2024 Dec 19;13(24):3546. doi: 10.3390/plants13243546 (PMC11680067; doi:10.3390/plants13243546)
Supplement: Supplementary file 1 [file plants-13-03546-s001.zip › Supplementary Figure 3.pdf]

Supplementary Figure S3 Identification results of F1 progenies of ‘HHQL’×‘HL’ and ‘HL’×‘HHQL’ cross combination.

SCoT-63

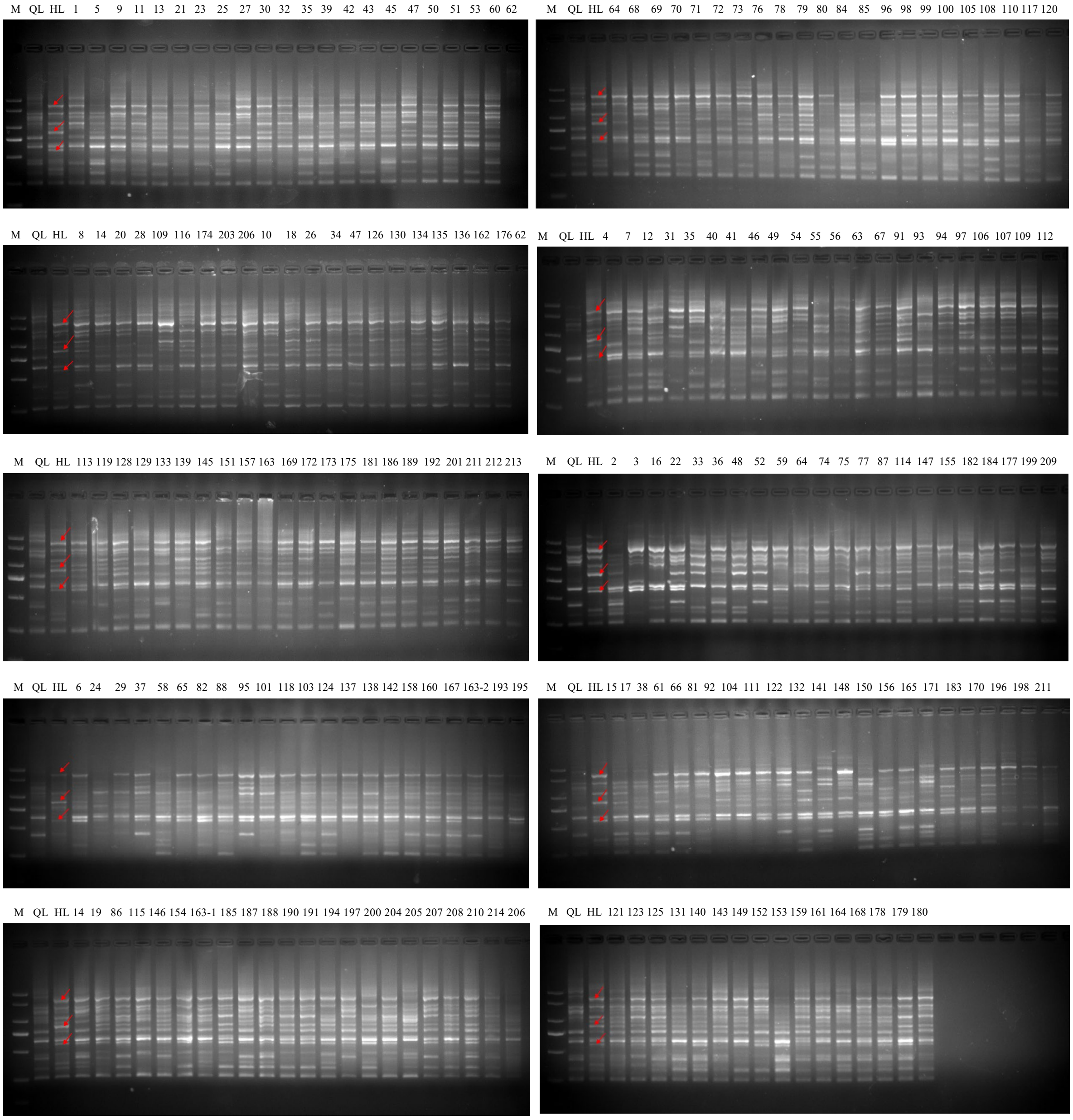

SCoT-12

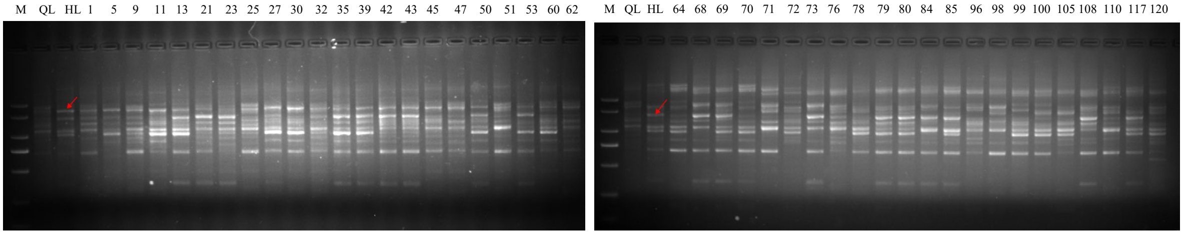

M QL HL 8 14 20 28 109 116 174 203 206 10 18 26 34 47 126 130 134 135 136 162 176 62

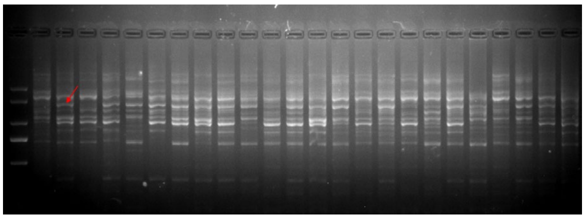

M QL HL 4 7 12 31 35 40 41 46 49 54 55 56 63 67 91 93 94 97 106 107 109 112

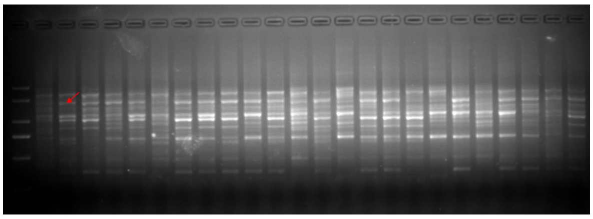

M QL HL 113 119 128 129 133 139 145 151 157 163 169 172 173 175 181 186 189 192 201 211 212 213

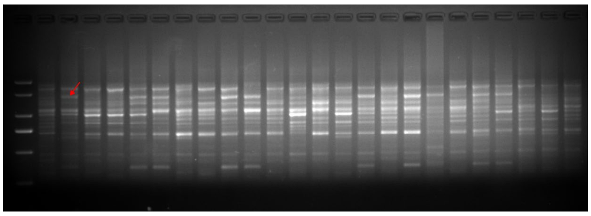

M QL HL 2 3 16 22 33 36 48 52 59 64 74 75 77 87 114 147 155 182 184 177 199 209

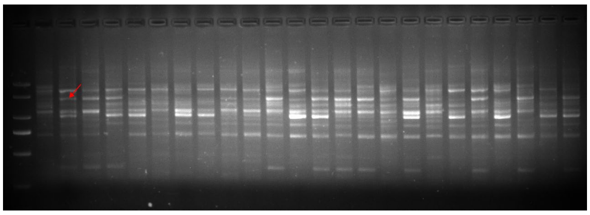

M QL HL 6 24 29 37 58 65 82 88 95 101 118 103 124 137 138 142 158 160 167 163-2 193 195

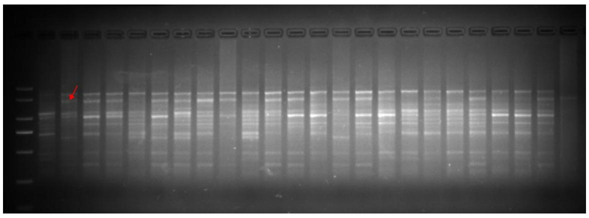

M QL HL 15 17 38 61 66 81 92 104 111 122 132 141 148 150 156 165 171 183 170 196 198 211

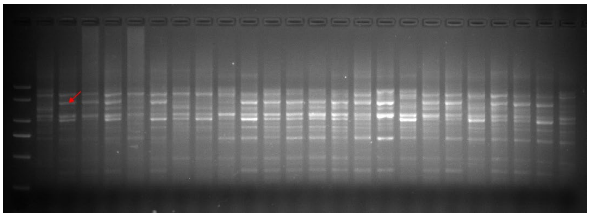

M QL HL 14 19 86 115 146 154 163-1 185 187 188 190 191 194 197 200 204 205 207 208 210 214 206

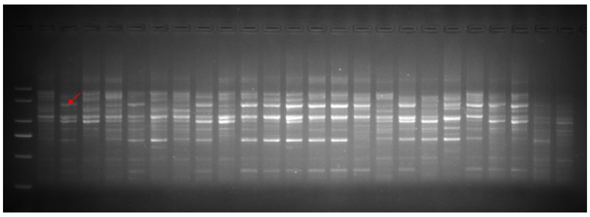

M QL HL 121 123 125 131 140 143 149 152 153 159 161 164 168 178 179 180

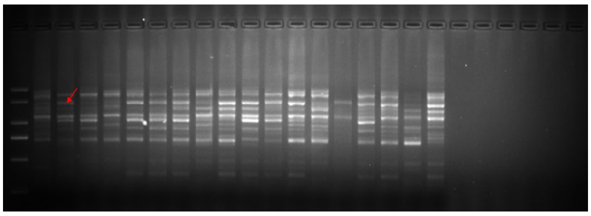

SRAP-M14E15

M QL HL 1 5 9 11 13 21 23 25 27 30 32 35 39 42 43 45 47 50 51 53 60 62

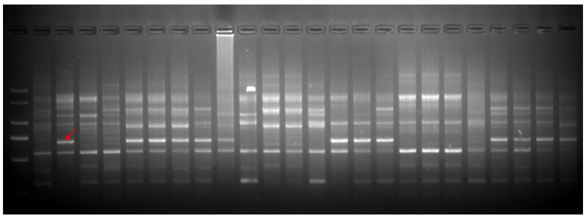

M QL HL 64 68 69 70 71 72 73 76 78 79 80 84 85 96 98 99 100 105 108 110 117 120

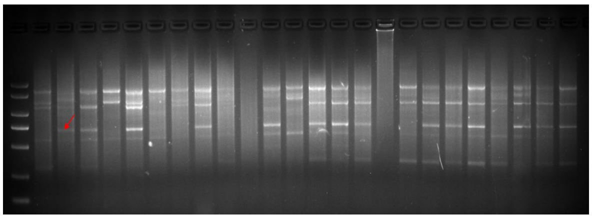

M QL HL 8 14 20 28 109 116 174 203 206 10 18 26 34 47 126 130 134 135 136 162 176 62

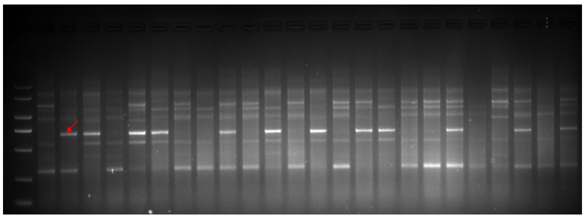

M QL HL 4 7 12 31 35 40 41 46 49 54 55 56 63 67 91 93 94 97 106 107 109 112

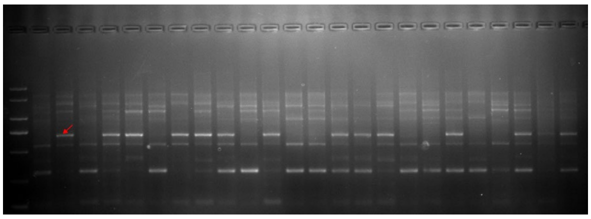

M QL HL 113 119 128 129 133 139 145 151 157 163 169 172 173 175 181 186 189 192 201 211 212 213

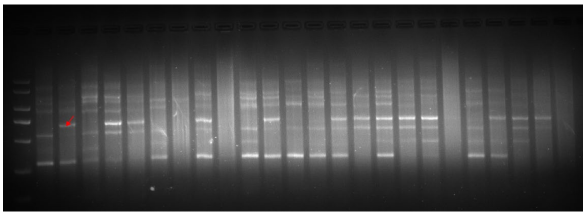

M QL HL 2 3 16 22 33 36 48 52 59 64 74 75 77 87 114 147 155 182 184 177 199 209

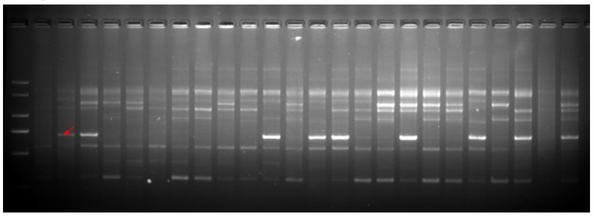

M QL HL 6 24 29 37 58 65 82 88 95 101 118 103 124 137 138 142 158 160 167 163-2 193 195

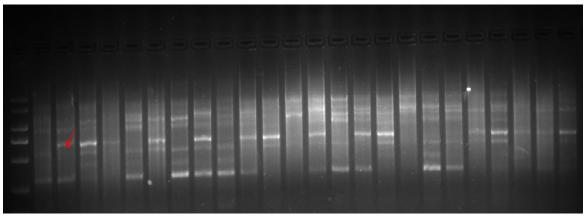

M QL HL 15 17 38 61 66 81 92 104 111 122 132 141 148 150 156 165 171 183 170 196 198 211

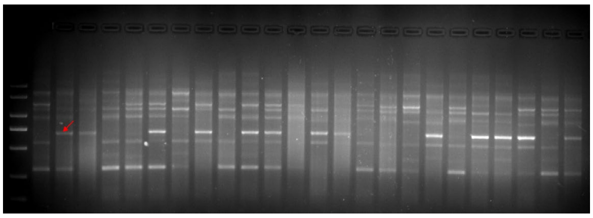

M QL HL 14 19 86 115 146 154 163-1 185 187 188 190 191 194 197 200 204 205 207 208 210 214 206

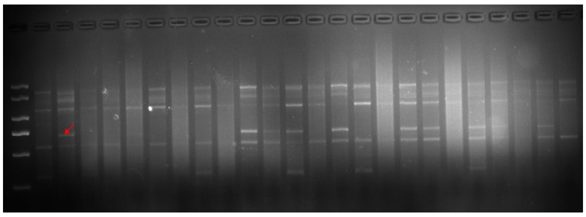

M QL HL 121 123 125 131 140 143 149 152 153 159 161 164 168 178 179 180

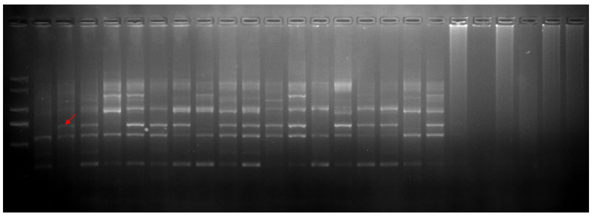

SRAP-M16E13

M QL HL 1 5 9 11 13 21 23 25 27 30 32 35 39 42 43 45 47 50 51 53 60 62

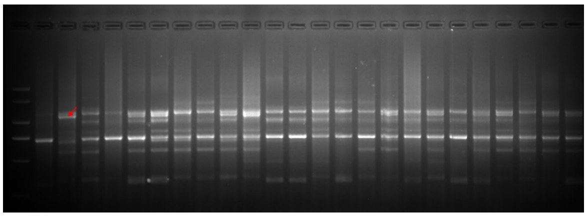

M QL HL 64 68 69 70 71 72 73 76 78 79 80 84 85 96 98 99 100 105 108 110 117 120

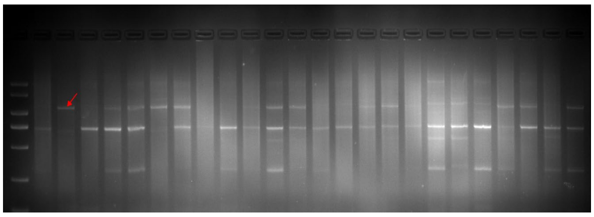

M QL HL 8 14 20 28 109 116 174 203 206 10 18 26 34 47 126 130 134 135 136 162 176 62

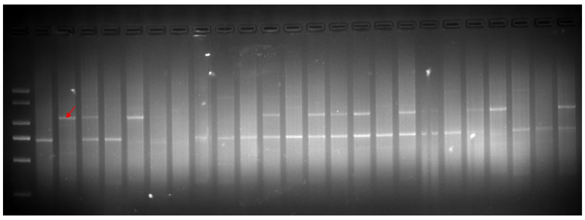

M QL HL 4 7 12 31 35 40 41 46 49 54 55 56 63 67 91 93 94 97 106 107 109 112

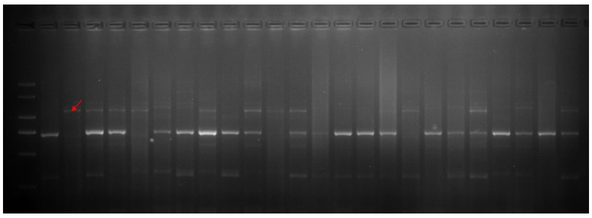

M QL HL 113 119 128 129 133 139 145 151 157 163 169 172 173 175 181 186 189 192 201 211 212 213

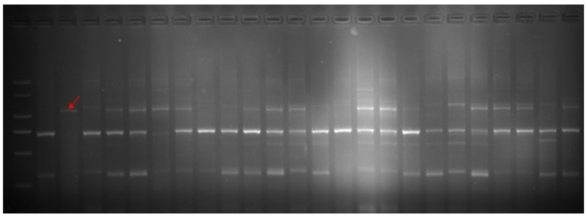

M QL HL 2 3 16 22 33 36 48 52 59 64 74 75 77 87 114 147 155 182 184 177 199 209

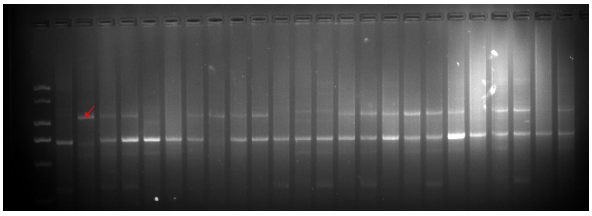

M QL HL 6 24 29 37 58 65 82 88 95 101 118 103 124 137 138 142 158 160 167 163-2 193 195

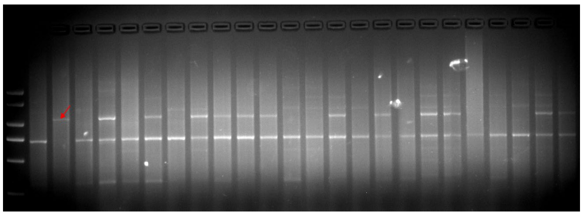

M QL HL 15 17 38 61 66 81 92 104 111 122 132 141 148 150 156 165 171 183 170 196 198 211

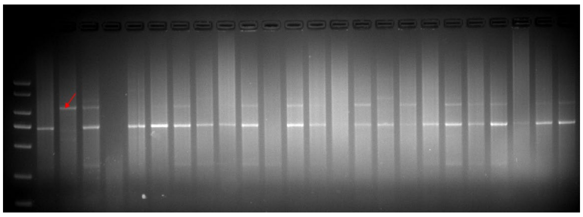

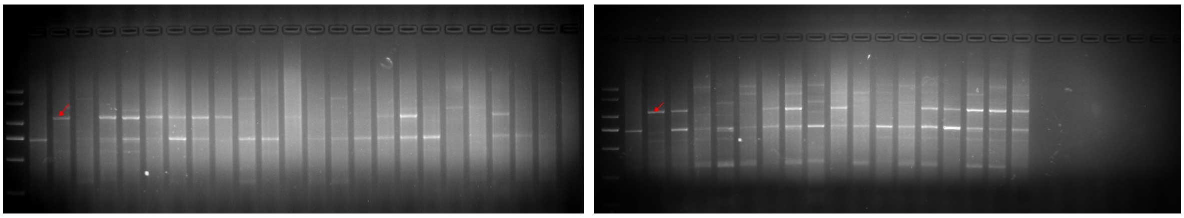

F1 progenies of ‘HHQL’×‘HL’ cross combination

SCoT-42

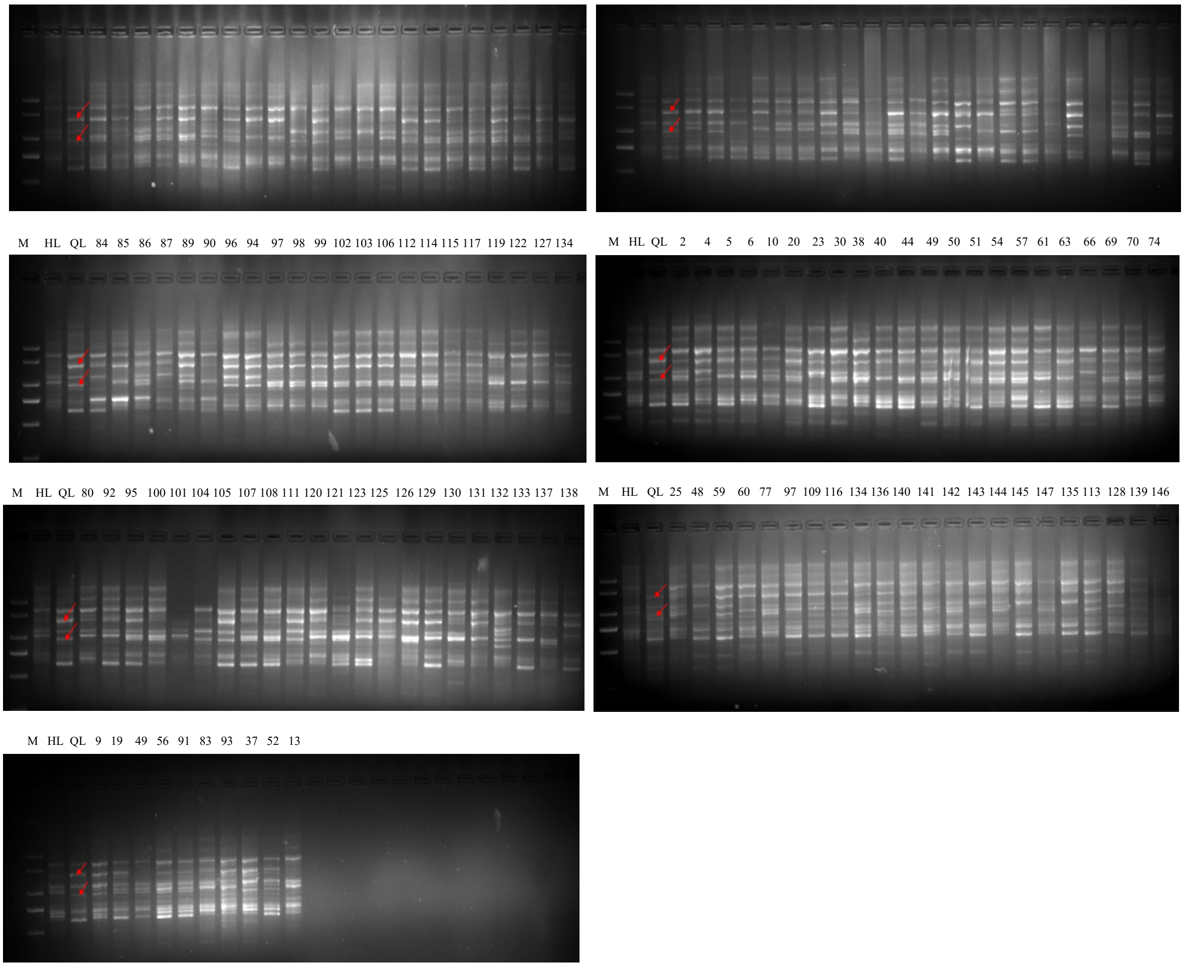

SCoT-21

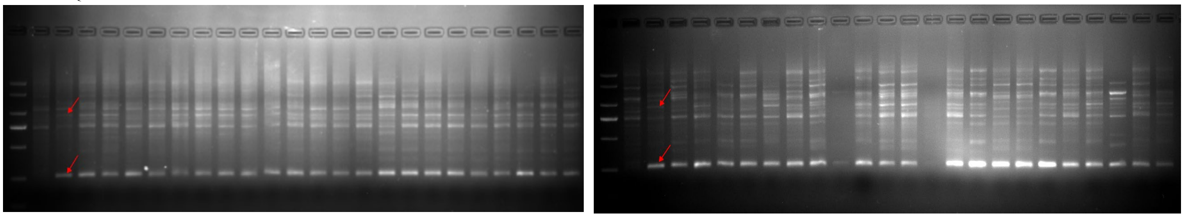

M HL QL 84 85 86 87 89 90 96 94 97 98 99 102 103 106 112 114 115 117 119 122 127 134

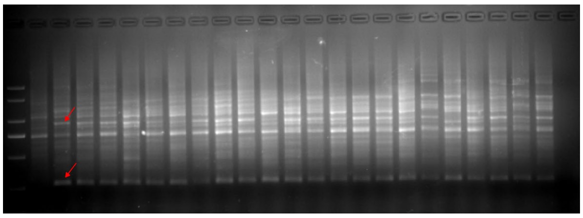

M HL QL 2 4 5 6 10 20 23 30 38 40 44 49 50 51 54 57 61 63 66 69 70 74

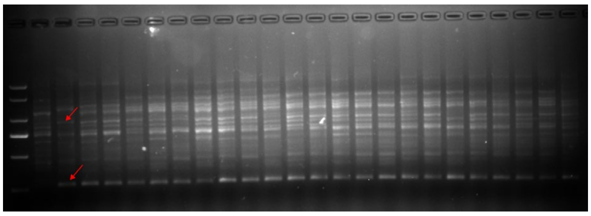

M HL QL 80 92 95 100 101 104 105 107 108 111 120 121 123 125 126 129 130 131 132 133 137 138

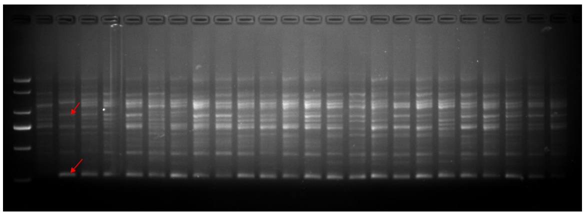

M HL QL 25 48 59 60 77 97 109 116 134 136 140 141 142 143 144 145 147 135 113 128 139 146

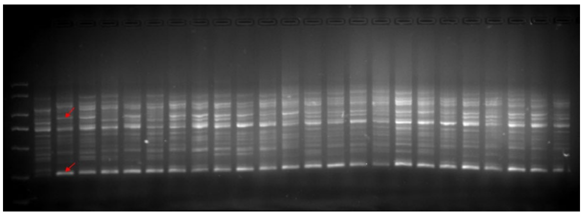

M HL QL 9 19 49 56 91 83 93 37 52 13

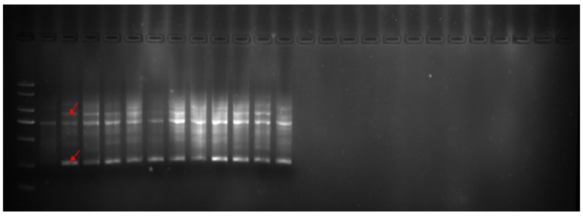

SRAP-M20E2

M HL QL 1 3 8 9 11 14 15 16 17 18 21 22 24 26 27 28 29 31 32 33 34 35

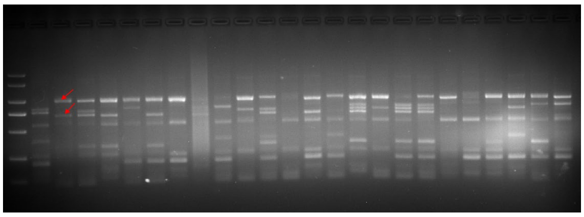

M HL QL 36 37 41 42 43 47 48 52 53 55 58 64 65 67 71 73 75 76 78 79 81 82

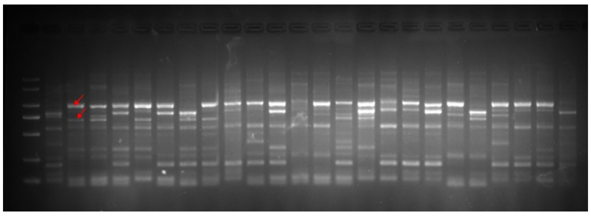

M HL QL 84 85 86 87 89 90 96 94 97 98 99 102 103 106 112 114 115 117 119 122 127 134

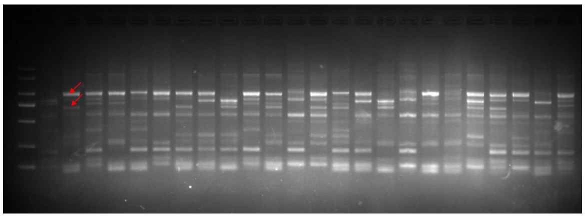

M HL QL 2 4 5 6 10 20 23 30 38 40 44 49 50 51 54 57 61 63 66 69 70 74

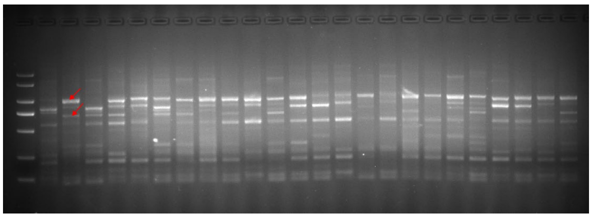

M HL QL 80 92 95 100 101 104 105 107 108 111 120 121 123 125 126 129 130 131 132 133 137 138

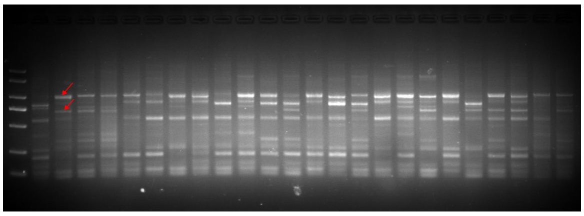

M HL QL 25 48 59 60 77 97 109 116 134 136 140 141 142 143 144 145 147 135 113 128 139 146

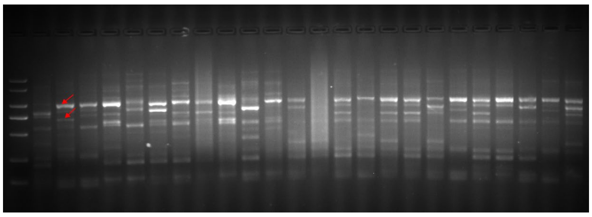

M HL QL 9 19 49 56 91 83 93 37 52 13

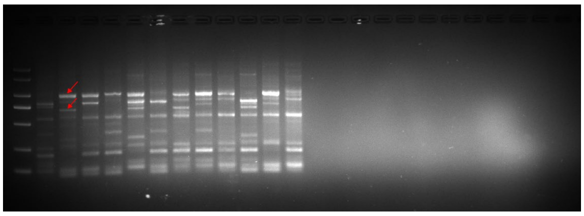

SRAP-M4E20

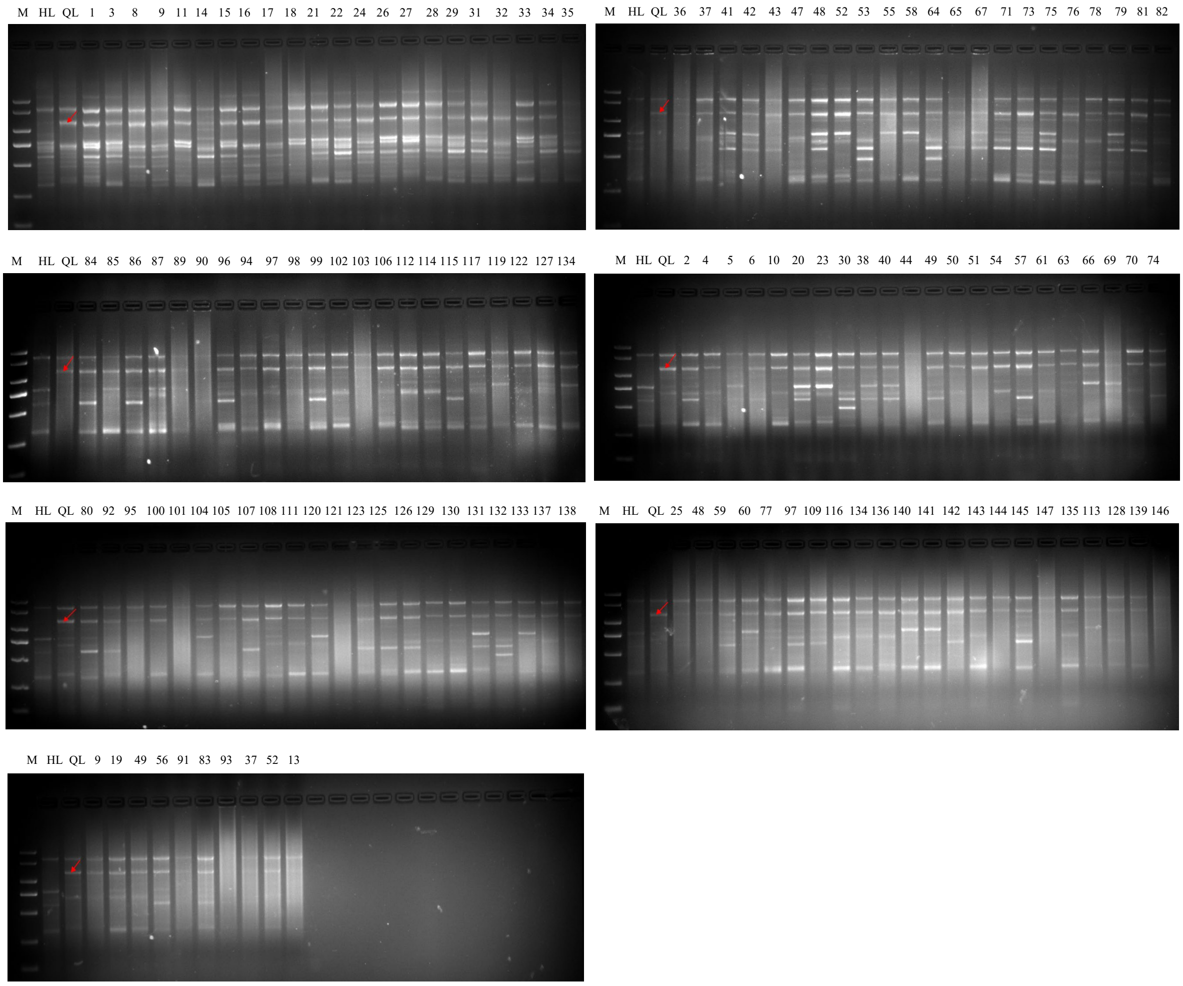

F1 progenies of ‘HL’×‘HHQL’ cross combination
