# Supplementary material for: Genetic Analyses of Flower, Fruit, and Stem Traits of Intergeneric Hybrids Between ‘Honghuagqinglong’ and ‘Heilong’ Pitayas
Source: Plants (Basel). 2024 Dec 19;13(24):3546. doi: 10.3390/plants13243546 (PMC11680067; doi:10.3390/plants13243546)
Supplement: Supplementary file 1 [file plants-13-03546-s001.zip › Supplementary Table 1.pdf]

Supplementary Table S1. Main flower and fruit traits of 'HHQL' and 'HL' pitayas.

Traits / Variety	Species	Petal color	Stigma split	Stigma-anther relative position	Peel color	Pulp color	Fruit weight (g)	TSS content (%)
**HHQL**	*Hylocereus*	red	present	higher	green	white	150.0a	19.0a
**HL**	*Seleniereus*	white	absent	equal	red	red	70.0b	16.0b
