# Supplementary material for: Genetic Analyses of Flower, Fruit, and Stem Traits of Intergeneric Hybrids Between ‘Honghuagqinglong’ and ‘Heilong’ Pitayas
Source: Plants (Basel). 2024 Dec 19;13(24):3546. doi: 10.3390/plants13243546 (PMC11680067; doi:10.3390/plants13243546)
Supplement: Supplementary file 1 [file plants-13-03546-s001.zip › Supplementary Table 2.pdf]

Supplementary Table S2. Comparison of stem traits of 'HHQL' and 'HL' pitayas.

Traits	'HHQL'	'HL'
Stem width (mm)	39.91±6.00	23.65±1.60
Stem edge thickness (mm)	4.85±0.61	10.79±1.30
Thorn No.	1.7±0.5	1.8±0.3
Thorn length (mm)	4.16±0.48	1.5±0.11
Distance between thorns (mm)	56.84±11.19	34.24±3.29
Infection degree of canker disease	3-4	0-1
