# Supplementary material for: Genetic Analyses of Flower, Fruit, and Stem Traits of Intergeneric Hybrids Between ‘Honghuagqinglong’ and ‘Heilong’ Pitayas
Source: Plants (Basel). 2024 Dec 19;13(24):3546. doi: 10.3390/plants13243546 (PMC11680067; doi:10.3390/plants13243546)
Supplement: Supplementary file 1 [file plants-13-03546-s001.zip › Supplementary Table 3.pdf]

Supplementary Table S3. SCoT primers used in this study.

Primer names	Sequences (5′-3′)	Primer names	Sequences (5′-3′)
**SCoT-12**	ACGACATGGCGACCAACG	**SCoT-56**	ACAATGGCTACCACTAGC
**SCoT-13**	ACGACATGGCGACCATCG	**SCoT-58**	ACAATGGCTACCACTAGG
**SCoT-19**	ACCATGGCTACCACCGGC	**SCoT-61**	CAACAATGGCTACCACCG
**SCoT-21**	ACGACATGGCGACCCACA	**SCoT-62**	ACCATGGCTACCACGGAG
**SCoT-36**	GCAACAATGGCTACCACC	**SCoT-63**	ACCATGGCTACCACGGGC
**SCoT-42**	ACCATGGCTACCACCGAT	**SCoT-64**	ACCATGGCTACCACGGTC
**SCoT-47**	ACAATGGCTACCACTGCC	**SCoT-67**	ACCATGGCTACCAGCGGC
**SCoT-49**	ACAATGGCTACCACTGCG	**SCoT-73**	CCATGGCTACCACCGGCT
