# Supplementary material for: Genetic Analyses of Flower, Fruit, and Stem Traits of Intergeneric Hybrids Between ‘Honghuagqinglong’ and ‘Heilong’ Pitayas
Source: Plants (Basel). 2024 Dec 19;13(24):3546. doi: 10.3390/plants13243546 (PMC11680067; doi:10.3390/plants13243546)
Supplement: Supplementary file 1 [file plants-13-03546-s001.zip › Supplementary Table 4.pdf]

Supplementary Table S4. 17 pairs of SRAP molecular marker primer.

No.	Pairs molecular marker primer	No.	Pairs molecular marker primer
**1**	me4em20	**10**	me14em20
**2**	me8em1	**11**	me15em12
**3**	me9em20	**12**	me15em16
**4**	me10em8	**13**	me15em20
**5**	me10em16	**14**	me16em13
**6**	me12em17	**15**	me19em19
**7**	me12em8	**16**	me20em2
**8**	me12em17	**17**	me21em15
**9**	me14em15		
