# Supplementary material for: Genetic Analyses of Flower, Fruit, and Stem Traits of Intergeneric Hybrids Between ‘Honghuagqinglong’ and ‘Heilong’ Pitayas
Source: Plants (Basel). 2024 Dec 19;13(24):3546. doi: 10.3390/plants13243546 (PMC11680067; doi:10.3390/plants13243546)
Supplement: Supplementary file 1 [file plants-13-03546-s001.zip › Supplementary Table 5.pdf]

**Supplementary Table S5.** SRAP primers used in this study.

| Forward primer names | Sequences | Reverse primer names | Sequences |
|---|---|---|---|
| me1 | TGAGTCCAAACCGGAAA | em1 | GACTGCGTACGAATTAAT |
| me2 | TGAGTCCAAACCGGAAT | em2 | GACTGCGTACGAATTAAC |
| me3 | TGAGTCCAAACCGGAAC | em3 | GACTGCGTACGAATTATG |
| me4 | TGAGTCCAAACCGGAAG | em4 | GACTGCGTACGAATTACG |
| me5 | TGAGTCCAAACCGGATA | em5 | GACTGCGTACGAATTAGC |
| me6 | TGAGTCCAAACCGGACA | em6 | GACTGCGTACGAATTTAG |
| me7 | TGAGTCCAAACCGGACT | em7 | GACTGCGTACGAATTTGA |
| me8 | TGAGTCCAAACCGGACC | em8 | GACTGCGTACGAATTTGC |
| me9 | TGAGTCCAAACCGGACG | em9 | GACTGCGTACGAATTTCA |
| me10 | TGAGTCCAAACCGGAGA | em10 | GACTGCGTACGAATTTCG |
| me11 | TGAGTCCAAACCGGAGC | em11 | GACTGCGTACGAATTCAA |
| me12 | TGAGTCCAAACCGGAGG | em12 | GACTGCGTACGAATTCAT |
| me13 | TGAGTCCAAACCGGTAG | em13 | GACTGCGTACGAATTCAC |
| me14 | TGAGTCCAAACCGGTTG | em14 | GACTGCGTACGAATTCAG |
| me15 | TGAGTCCAAACCGGTCA | em15 | GACTGCGTACGAATTCTA |
| me16 | TGAGTCCAAACCGGTGT | em16 | GACTGCGTACGAATTCTT |
| me17 | TGAGTCCAAACCGGTGC | em17 | GACTGCGTACGAATTCTC |
| me18 | TGAGTCCAAACCGGCAG | em18 | GACTGCGTACGAATTCTG |
| me19 | TGAGTCCAAACCGGCTA | em19 | GACTGCGTACGAATTCCA |
| me20 | TGAGTCCAAACCGGGAC | em20 | GACTGCGTACGAATTCGA |
| me21 | TGAGTCCAAACCGGGTA | em21 | GACTGCGTACGAATTCGG |
| | | em22 | GACTGCGTACGAATTGAT |
| | | em23 | GACTGCGTACGAATTCAC |
| | | em24 | GACTGCGTACGAATTCAG |
