# Supplementary material for: Genetic Analyses of Flower, Fruit, and Stem Traits of Intergeneric Hybrids Between ‘Honghuagqinglong’ and ‘Heilong’ Pitayas
Source: Plants (Basel). 2024 Dec 19;13(24):3546. doi: 10.3390/plants13243546 (PMC11680067; doi:10.3390/plants13243546)
Supplement: Supplementary file 1 [file plants-13-03546-s001.zip › Supplementary Table 6.pdf]

Supplementary Table S6. Disease index of pitaya canker disease.

Level	Disease description
0	No pitaya canker disease
1	There are few pitaya canker disease and lesion area is approximately 0.5-2 mm
2	Lesion area is 5-15%
3	Lesion area is 15-30%
4	Lesion area is 30-60%
5	Lesion area is higher than 60%
