# Supplementary material for: Genetic Analyses of Flower, Fruit, and Stem Traits of Intergeneric Hybrids Between ‘Honghuagqinglong’ and ‘Heilong’ Pitayas
Source: Plants (Basel). 2024 Dec 19;13(24):3546. doi: 10.3390/plants13243546 (PMC11680067; doi:10.3390/plants13243546)
Supplement: Supplementary file 1 [file plants-13-03546-s001.zip › Supplementary Table 7.pdf]

Supplementary Table S7. The AIC values of flower main traits of F_1 progenies from 'HHQL' × 'HL' and 'HL' × 'HHQL' cross combinations under different genetic models.

Genetic model<br>Flower traits	Flower length		Perianth width		Calyx tube width		Distance between stigma and anther		Petal color		No. of petals		No. of stigma lobe	
	Q×H	H×Q	Q×H	H×Q	Q×H	H×Q	Q×H	H×Q	Q×H	H×Q	Q×H	H×Q	Q×H	H×Q
0MG	**611.4108**	410.97	186.2661	148.006	-1.8183	**-24.2216**	354.0023	177.7085	491.4351	289.8298	641.185	409.5061	648.2448	407.833
1MG-AD	613.6328	413.1385	186.9737	137.8282	-2.8235	-21.6199	314.4846	152.3696	**371.6536**	248.4603	641.0626	410.3648	641.5048	409.4562
1MG-A	612.1792	412.1074	185.054	137.3703	-3.9556	-23.594	325.2127	161.9636	436.1686	258.5648	**638.6906**	**408.3598**	**640.8641**	407.9384
1MG-EAD	614.4976	414.6917	189.9468	139.893	-2.5157	-20.5851	331.614	169.098	399.866	254.2165	643.091	411.4244	643.2303	410.4933
1MG-NCD	615.3671	414.6498	190.1808	140.6655	1.0177	-20.5953	331.575	163.9402	435.1437	266.0884	644.0629	411.7562	642.9352	410.5886
2MG-ADI	626.1317	426.6187	196.5997	147.8048	8.716	-8.6019	330.291	175.1311	439.0845	267.5757	655.8444	422.5595	651.9189	421.5981
2MG-AD	**611.6487**	**402.3171**	**182.4063**	**136.5863**	-1.4603	**-24.8308**	**-909.3835**	-569.9392	**369.7245**	**224.917**	640.7653	**409.4973**	641.3372	**393.2549**
2MG-A	612.3986	414.7646	190.2248	138.8392	-2.9507	-20.3876	314.0057	154.0914	392.9999	253.4791	**639.7262**	410.7734	**639.8956**	407.8995
2MG-EA	**610.8403**	409.2928	**180.9867**	**134.6971**	**-6.9657**	**-24.8485**	-558.7752	**-694.4596**	409.2857	246.2891	**638.4009**	**405.5957**	**639.4881**	407.4531
2MG-CD	615.4193	414.974	190.3232	152.0061	2.1866	-20.2174	358.0021	181.7069	495.4343	293.8277	645.189	413.5056	652.2459	411.8382
2MG-EAD	613.4195	412.9741	188.2777	150.006	0.1865	-22.2173	356.002	179.7069	493.4343	291.8277	643.189	411.5056	650.246	409.8382

Note: The AIC values of candidate genetic model were highlighted in bold font.
