# Supplementary material for: Genetic Analyses of Flower, Fruit, and Stem Traits of Intergeneric Hybrids Between ‘Honghuagqinglong’ and ‘Heilong’ Pitayas
Source: Plants (Basel). 2024 Dec 19;13(24):3546. doi: 10.3390/plants13243546 (PMC11680067; doi:10.3390/plants13243546)
Supplement: Supplementary file 1 [file plants-13-03546-s001.zip › Supplementary Table 8.pdf]

Supplementary Table S8. Optimal model for flower main traits of F1 progenies from 'HHQL' × 'HL' and 'HL' × 'HHQL' cross combinations using suitable test.

Traits	Cross combination	Model	AIC	U_1^2	U_2^2	U_3^2	nW^2	D_n
Flower length	Q×H	2MG-EA	610.8403	0.0001(0.9938)	0.0002(0.9896)	0.0067(0.9348)	0.0171(0.999)	0.0308(0.9988)
	H×Q	2MG-AD	402.3171	0.0022(0.9627)	0.0007(0.9783)	0.0052(0.9425)	0.0094(1.0001)	0.0311(1)
Perianth width	Q×H	2MG-EA	180.9867	0.0087(0.9257)	0.0021(0.9635)	0.0318(0.8586)	0.0277(0.9832)	0.0542(0.7713)
	H×Q	2MG-EA	134.6971	0.0071(0.9329)	0.02(0.8876)	0.0573(0.8107)	0.0164(0.9992)	0.0427(0.9931)
Calyx tube width	Q×H	2MG-EA	-6.9657	0.0006(0.9811)	0.0005(0.9829)	0(0.9952)	0.019(0.9978)	0.0308(0.9989)
	H×Q	2MG-EA	-24.8485	0.0012(0.9723)	0.0001(0.9936)	0.0277(0.8679)	0.0212(0.9957)	0.0435(0.9916)
Distance between stigma and anther	Q×H	2MG-AD	-909.3835	0.0026(0.9595)	0.0003(0.9863)	0.0163(0.8983)	0.0178(0.9986)	0.0473(0.8903)
	H×Q	2MG-EA	-694.4596	0.0084(0.9271)	0.0002(0.9901)	0.0928(0.7607)	0.0568(0.8357)	0.0981(0.2914)
Petal color	Q×H	2MG-AD	369.7245	0(0.9969)	0.0005(0.9819)	0.0058(0.9395)	0.038(0.9431)	0.0509(0.867)
	H×Q	2MG-AD	224.917	0.0017(0.9674)	0(0.995)	0.0337(0.8543)	0.0163(0.9993)	0.0395(0.997)
No. of petals	Q×H	2MG-EA	638.4009	0.0003(0.9862)	0.0016(0.9679)	0.0089(0.925)	0.0315(0.9711)	0.0559(0.7363)
	H×Q	2MG-EA	405.5957	0.0001(0.9908)	0.0032(0.955)	0.0328(0.8563)	0.0135(0.9998)	0.0401(0.9971)
No. of stigma lobes	Q×H	2MG-EA	639.4881	0.001(0.9752)	0.0018(0.9666)	0.0022(0.9624)	0.0195(0.9974)	0.032(0.9979)
	H×Q	2MG-AD	393.2549	0.0031(0.9553)	0.0015(0.9693)	0.004(0.9498)	0.0175(0.9988)	0.0435(0.988)
