# Supplementary material for: Genetic Analyses of Flower, Fruit, and Stem Traits of Intergeneric Hybrids Between ‘Honghuagqinglong’ and ‘Heilong’ Pitayas
Source: Plants (Basel). 2024 Dec 19;13(24):3546. doi: 10.3390/plants13243546 (PMC11680067; doi:10.3390/plants13243546)
Supplement: Supplementary file 1 [file plants-13-03546-s001.zip › Supplementary Table 9.pdf]

Supplementary Table S9. The AIC values of fruit main traits of 'HHQL' × 'HL' cross combinations under different genetic models.

Model	Fruit weight	Fruit longitudinal diameter	Fruit transverse diameter	Fruit shape index	No. of scales	Basal width of middle scales	Pulp hardness	TSS content	Fruit top cavity	Edible rate	Peel weight	Peel thickness	Peel color	Pulp color
0MG	1458.406	510.1181	287.5049	47.5663	1020.326	94.3705	-296.8103	631.1985	171.3458	-316.2293	1277.715	-283.2747	1016.833	1117.083
1MG-AD	1444.681	510.3786	**286.3358**	43.1255	1018.824	52.8448	-301.6844	633.1522	157.6479	-321.8076	1270.536	-304.1261	969.8725	**725.8699**
1MG-A	1442.979	511.3181	287.5121	46.2632	**1016.837**	50.8318	**-303.6848**	**631.1503**	157.2597	**-322.7073**	1269.052	-301.9176	979.055	**734.2952**
1MG-EAD	1453.081	513.3406	289.5178	47.9419	1020.108	77.7438	-298.7513	633.2593	164.3054	-315.5639	1274.542	-297.8165	992.0527	735.888
1MG-NCD	1455.882	513.6734	289.4922	51.5224	1022.688	97.2273	-295.9482	633.8634	162.0314	-312.227	1276.332	-297.2219	997.3541	948.2493
2MG-ADI	1463.461	525.3021	301.4841	59.574	1030.727	60.9212	-289.9882	645.2734	171.0354	-309.2804	1280.219	-301.2965	983.3385	908.4748
2MG-AD	1444.884	502.6483	293.5002	**38.162**	1019.529	44.5718	-298.6729	637.1275	**-537.078**	**-324.2697**	**1263.052**	**-304.9506**	**962.6382**	**734.9321**
2MG-A	**1440.269**	513.6151	289.5483	**37.1973**	**1016.918**	**36.0351**	-300.8466	633.1286	157.9593	-321.2507	**1266.452**	**-306.769**	**967.2196**	829.6816
2MG-EA	**1443.458**	**497.2864**	**285.6353**	39.8131	**1017.762**	**40.9096**	**-303.5709**	**629.8581**	-376.0634	**-322.847**	**1266.139**	**-306.3519**	**964.4495**	824.2671
2MG-CD	1462.407	514.1231	289.6457	51.5725	1024.329	98.3693	-292.8093	633.2926	175.3461	-315.5411	1281.716	-279.2744	1020.833	1121.083
2MG-EAD	1460.407	512.1231	287.6459	49.5727	1022.329	96.3693	-294.8092	**631.3702**	173.3462	-315.8853	1279.716	-281.2746	1018.833	1119.083
