# Supplementary material for: Genetic Analyses of Flower, Fruit, and Stem Traits of Intergeneric Hybrids Between ‘Honghuagqinglong’ and ‘Heilong’ Pitayas
Source: Plants (Basel). 2024 Dec 19;13(24):3546. doi: 10.3390/plants13243546 (PMC11680067; doi:10.3390/plants13243546)
Supplement: Supplementary file 1 [file plants-13-03546-s001.zip › Supplementary Table 10.pdf]

Supplementary Table S10. The AIC values of fruit main traits of 'HL' × 'HHQL' cross combinations under different genetic models.

Model	Fruit weight	Fruit longitudinal diameter	Fruit transverse diameter	Fruit shape index	No. of scales	Basal width of middle scales	Flesh hardness	TSS content	Fruit top cavity	Edible rate	Peel weight	Peel thickness	Peel color	Pulp color
0MG	980.1219	332.1103	190.5761	7.4922	679.3288	50.2744	-219.5673	450.1629	80.4374	-181.7909	823.7246	-186.6419	711.8992	773.6948
1MG-AD	964.4402	328.8681	182.35	9.8324	675.742	42.2839	-220.5756	445.9901	61.4039	-179.957	818.8714	-194.8365	**674.7539**	**585.9142**
1MG-A	963.2678	330.9725	180.4052	8.0605	675.4109	40.2912	**-222.5576**	444.1047	**60.0455**	-180.5171	**816.9012**	-196.2782	694.3032	620.3712
1MG-EAD	971.537	335.0768	186.72	11.2668	678.1124	45.8105	-218.5337	447.0515	71.8713	-178.853	821.5974	-189.196	683.0181	**588.5993**
1MG-NCD	976.4603	335.1599	190.9179	11.4963	676.3772	49.994	-217.0894	454.1612	65.1352	-177.7881	822.2367	-189.292	690.6914	720.3599
2MG-ADI	984.1343	332.9156	194.5021	4.2523	688.3036	57.1778	-208.8067	458.8401	74.6005	-168.2464	828.0065	-191.5104	685.3465	638.9638
2MG-AD	**957.6693**	**325.6944**	**169.8638**	-4.3613	**669.8228**	41.4421	-216.6822	**440.2321**	62.976	-179.8779	**814.4208**	**-759.0124**	**669.0484**	**586.531**
2MG-A	964.5901	**326.1319**	182.4011	**-293.5169**	675.0515	40.6679	-219.9727	444.9629	**60.9079**	-178.0475	818.8947	-202.1965	679.2879	689.554
2MG-EA	**961.4811**	**325.8427**	182.668	8.1177	**669.5107**	**29.5598**	**-222.449**	**439.3732**	**58.6764**	**-184.3132**	**815.7682**	-196.9494	677.0438	626.6353
2MG-CD	984.1207	336.1102	194.5764	11.384	683.3282	54.2724	-215.568	447.4416	84.4354	-178.5785	827.7238	-182.6437	715.8972	777.6941
2MG-EAD	982.1208	334.1103	192.5764	9.3875	681.3283	52.2722	-217.5679	445.6429	82.4355	-180.3655	825.7238	-184.6437	713.8972	775.6941
