# Supplementary material for: Genetic Analyses of Flower, Fruit, and Stem Traits of Intergeneric Hybrids Between ‘Honghuagqinglong’ and ‘Heilong’ Pitayas
Source: Plants (Basel). 2024 Dec 19;13(24):3546. doi: 10.3390/plants13243546 (PMC11680067; doi:10.3390/plants13243546)
Supplement: Supplementary file 1 [file plants-13-03546-s001.zip › Supplementary Table 11.pdf]

Supplementary Table S11. Optimal model for fruit main traits of F_1 progenies from 'HHQL' × 'HL' cross combination using suitable test.

Fruit traits	Model	AIC	U_1^2	U_2^2	U_3^2	nW^2	Dn
Fruit weight	2MG-A	1440.269	0.0008(0.9775）	0.0001(0.9937）	0.0199(0.8878）	0.0143(0.9997）	0.0278(0.9998）
	2MG-EA	1443.458	0.0006(0.9812）	0.001(0.9745）	0.0481(0.8263）	0.0328(0.966）	0.0564(0.7265）
Fruit longitudinal diameter	2MG-EA	497.2864	0.0002(0.9894）	0(0.9967）	0.0012(0.9723）	0.013(0.9999）	0.0248(1）
Fruit transverse diameter	1MG-AD	286.3358	0(0.9979）	0(0.9984）	0(0.9984）	0.0137(0.9668）	0.0287(0.9997）
	2MG-EA	285.6353	0.0007(0.9784）	0.0002(0.9881）	0.0271(0.8692）	0.0346(0.9586）	0.0434(0.9399）
Fruit shape index	2MG-AD	38.162	0.0029(0.9573）	0.0037(0.9515）	0.0013(0.9715）	0.0103(1）	0.0259(1）
	2MG-A	37.1973	0.0042(0.9485）	0.0035(0.953）	0.0002(0.9885）	0.0112(1）	0.0261(1）
Number of scales	1MG-A	1016.837	0.0004(0.9844）	0.0009(0.9757）	0.0021(0.9633）	0.017(0.999）	0.0386(0.9788）
	2MG-A	1016.918	0.042790.8362)	0.0402(0.8412）	0(0.9993）	0.0152(0.9996）	0.0357(0.9911）
	2MG-EA	1017.762	0.01259(0.9111）	0.0113(0.9152）	0(0.9948）	0.0195(0.9975）	0.0359(0.9905）
Basal width of middle scales	2MG-A	36.0351	0.1693(0.6808)	0.2143(0.6434)	0.0668(0.796)	0.0263(0.9867)	0.0306(0.999)
	2MG-EA	40.9096	0.0043(0.9479)	0.0002(0.99)	0.0411(0.8394)	0.0212(0.9958)	0.0383(0.9802)
Flesh hardness	1MG-A	-303.6848	0.0013(0.9714)	0.0037(0.9513)	0.0111(0.916)	0.0351(0.9567)	0.0461(0.9092)
	2MG-EA	-303.5709	0.0006(0.9798)	0.0024(0.9611)	0.0094(0.9227)	0.0344(0.9594)	0.0459(0.912）
TSS content	1MG-A	631.1503	0.0001(0.9931）	0.0001(0.9943）	0(0.996）	0.0153(0.9996）	0.034(0.995）
	2MG-EA	629.8581	0.0000(0.9957）	0.0001(0.9919）	0.0004(0.9843）	0.0156(0.9995）	0.0331(0.9965）
	2MG-EAD	631.3702	0.0005(0.9814）	0.0005(0.9821）	0(0.9993）	0.017(0.999）	0.0345(0.9942）
Fruit top cavity	2MG-AD	-537.078	0.002(0.9647）	0.0028(0.9577）	0.0016(0.9677）	0.0186(0.9981）	0.0351(0.9926）

	1MG-A	-322.7073	0.0021(0.9637)	0.0162(0.8987)	0.1108(0.7393)	0.0387(0.9397)	0.0457(0.9123）
Edible rate	2MG-AD	-324.2697	0(0.9988）	0(0.9954)	0.0003(0.9862)	0.0324(0.9676)	0.0424(0.9503)
	2MG-EA	-322.847	0.0046(0.9458）	0.0205(0.8862)	0.0955(0.7573)	0.0383(0.9418)	0.0453(0.9183)
	2MG-AD	1263.052	0.0001(0.9925）	0.0001(0.9908）	0.0001(0.9921）	0.0075(1.0012）	0.0227(1）
Peel weight	2MG-A	1266.452	0.0221(0.8818）	0.0241(0.8766）	0.002(0.964）	0.0133(0.9999）	0.03(0.9993）
	2MG-EA	1266.139	0.0012(0.9719）	0(0.9987）	0.0205(0.8863）	0.0164(0.9992）	0.0334(0.9962）
	2MG-AD	-304.9506	0.0002(0.9891）	0.0006(0.9808）	0.0019(0.9652）	0.0364(0.9505）	0.0462(0.9055）
Peel thickness	2MG-A	-306.769	0.0458(0.8306）	0.0601(0.8063）	0.0231(0.8791）	0.0433(0.9157）	0.0503(0.8422）
	2MG-EA	-306.3519	0.0194(0.8891）	0.0224(0.8809）	0.0035(0.953）	0.0471(0.8936）	0.0571(0.7122）
	2MG-AD	962.6382	0.0006(0.98）	0.0009(0.976）	0.0005(0.9813）	0.0085(1.0003）	0.0223(1）
Peel color	2MG-A	967.2196	0.1504(0.6981）	0.1962(0.6578）	0.0726(0.7876）	0.044(0.9115）	0.0462(0.9147）
	2MG-EA	964.4495	0.0032(0.9546）	0.0091(0.9239）	0.0261(0.8717）	0.0257(0.9881）	0.0369(0.9887）
	1MG-AD	725.8699	0.0149(0.9029）	0.0298(0.863）	0.0475(0.8276）	0.02(0.997）	0.0371(0.988）
Pulp color	1MG-A	734.2952	0.0736(0.7861）	0.0377(0.8461）	0.0754(0.7837）	0.0547(0.848）	0.0479(0.8906）
	2MG-AD	734.9321	0.0035(0.9527）	0.0114(0.9148）	0.0393(0.8428）	0.0274(0.9839）	0.0373(0.9874）

*The *P* values of each trait are shown in brackets.
