# Supplementary material for: Genetic Analyses of Flower, Fruit, and Stem Traits of Intergeneric Hybrids Between ‘Honghuagqinglong’ and ‘Heilong’ Pitayas
Source: Plants (Basel). 2024 Dec 19;13(24):3546. doi: 10.3390/plants13243546 (PMC11680067; doi:10.3390/plants13243546)
Supplement: Supplementary file 1 [file plants-13-03546-s001.zip › Supplementary Table 12.pdf]

Supplementary Table S12. Optimal model for fruit main traits of F_1 progenies from 'HL' × 'HHQL' cross combinations using suitable test.

Fruit traits	Model	AIC	U_1^2	U_2^2	U_3^2	nW^2	Dn
Fruit weight	2MG-AD	957.6693	0.0144(0.9046）	0.008(0.9287)	0.0113(0.9152)	0.0112(1)	0.0293(1)
	2MG-EA	961.4811	0.0007(0.9782)	0.0097(0.9217)	0.2492(0.6176)	0.0297(0.9772)	0.0521(0.9492)
Fruit longitudinal diameter	2MG-AD	325.6944	0.001(0.9751）	0.0012(0.9727）	0.0003(0.9873）	0.0193(0.9976）	0.0427(0.9932）
	2MG-A	326.1319	0.0125(0.911）	0.0295(0.8635）	0.0649(0.7989）	0.0249(0.9898）	0.043(0.9926）
	2MG-EA	325.8427	0.0029(0.9573）	0.0004(0.9834）	0.0154(0.9011）	0.0362(0.9518）	0.0509(0.9582）
Fruit transverse diameter	2MG-AD	169.8638	0.0014(0.9703）	0.0015(0.9688）	0.0002(0.9902）	0.0121(1）	0.0349(0.9997）
Fruit shape index	2MG-A	-293.5169	0.0017(0.9674）	0.0001(0.9907）	0.0124(0.9113）	0.0192(0.9977）	0.0418(0.9949）
Number of scales	2MG-AD	669.8228	0.0004(0.9841）	0.0007(0.9782）	0.001(0.9745）	0.01(1）	0.0282(1）
	2MG-EA	669.5107	0(0.999）	0.0008(0.9781）	0.0131(0.9089）	0.0181(0.9985）	0.0381(0.9987）
Basal width of middle scales	2MG-EA	29.5598	0.0103(0.9192）	0.0404(0.8408）	0.1687(0.6813）	0.0302(0.9754）	0.0505(0.9509）
Flesh hardness	1MG-A	-222.5576	0.0001(0.9933）	0.0004(0.9833）	0.0026(0.9591）	0.0351(0.9567）	0.0583(0.8861）
	2MG-EA	-222.449	0.0003(0.9862）	0.0011(0.9739）	0.0041(0.9491）	0.0349(0.9576）	0.0585(0.8832）
TSS content	2MG-AD	440.2321	0.0735(0.7863）	0.0533(0.8175）	0.0161(0.8991）	0.0245(0.9905）	0.0528(0.9433）
	2MG-EA	439.3732	0.0567(0.8118）	0.0178(0.8939）	0.151(0.6975）	0.023(0.9933）	0.0468(0.9809）
Top cavity of fruit stalk	1MG-A	60.0455	0.0215(0.8833）	0.0277(0.8678）	0.0094(0.9226）	0.0316(0.9705）	0.054(0.9321）
	2MG-A	60.9079	0.0175(0.8947）	0.0213(0.884）	0.005(0.9436）	0.0205(0.9965）	0.0434(0.9917）
	2MG-EA	58.6764	0.0268(0.8699）	0.0289(0.865）	0.0021(0.9634）	0.024(0.9915）	0.044(0.9902）
Edible rate	2MG-EA	-184.3132	0.0146(0.9038）	0.0068(0.9343）	0.0191(0.89）	0.0264(0.9865）	0.0498(0.9568）
Peel weight	1MG-A	816.9012	0.0011(0.9741）	0.0008(0.9778）	0.0002(0.9884）	0.028(0.9822)	0.0542(0.9182)
	2MG-AD	814.4208	0(0.9995）	0.0004(0.9845）	0.0057(0.9397）	0.0167(0.9991）	0.0474(0.9717)
	2MG-EA	815.7682	0.001(0.9746）	0(0.9964）	0.0111(0.916）	0.0239(0.9917）	0.051(0.9473)
Peel thickness	2MG-AD	-759.0124	0.0168(0.8967）	0.0175(0.8947）	0.0007(0.9786)	0.0432(0.9158)	0.0838(0.4838）
Peel color	1MG-AD	674.7539	0.0031(0.9554)	0(0.9974)	0.0415(0.8387）	0.0302(0.9754）	0.0472(0.9737）
	2MG-AD	669.0484	0.001(0.9746）	0(0.9956)	0.0103(0.9191）	0.0234(0.9925）	0.0441(0.9868）
Pulp color	1MG-AD	585.9142	0.0223(0.8813）	0.0051(0.9433)	0.0862(0.7691)	0.0263(0.9866)	0.0497(0.9587)
	1MG-EAD	588.5993	0.015(0.9025)	0.0006(0.9805)	0.1417(0.7066)	0.0266(0.986)	0.0475(0.9723）
	2MG-AD	586.531	0.0005(0.983）	0.0003(0.986)	0.0002(0.99)	0.0075(1.001)	0.0253(1)

*The *P* values of each trait are shown in brackets.
