# Supplementary material for: Genetic Analyses of Flower, Fruit, and Stem Traits of Intergeneric Hybrids Between ‘Honghuagqinglong’ and ‘Heilong’ Pitayas
Source: Plants (Basel). 2024 Dec 19;13(24):3546. doi: 10.3390/plants13243546 (PMC11680067; doi:10.3390/plants13243546)
Supplement: Supplementary file 1 [file plants-13-03546-s001.zip › Supplementary Table 13.pdf]

Supplementary Table S13. The AIC values of stem traits from 'HHQL' × 'HL' and 'HL' × 'HHQL' cross combinations under different genetic models.

Model	Stem width		Stem edge thickness		No. of thorns		Length of thorns		Distance between thorns	
	Q×H	H×Q	Q×H	H×Q	Q×H	H×Q	Q×H	H×Q	Q×H	H×Q
0MG	1377.388	902.1005	1122.399	686.2437	599.28	376.395	740.7789	469.3612	1538.816	985.6013
1MG-AD	1377.944	902.003	1094.959	646.1791		-3165.081	673.9756	440.9772	1534.746	985.034
1MG-A	1377.115	902.6609	1099.537	645.0442	362.9629	228.3466	689.9822	441.9137	1536.416	983.0809
1MG-EAD	1380.634	905.5975	1101.624	668.7223	-4609.794	-3281.205	685.5565	444.9755	1538.498	986.4022
1MG-NCD	1380.908	905.6853	1095.717	681.0704	589.6298	372.6953	691.1343	456.2554	1537.69	988.3791
2MG-ADI	1391.331	916.5047	1107.587	663.0373	581.1751	72.9595	680.3685	462.7658	1548.624	999.0242
2MG-AD	1367.768	891.2013	1077.813	635.3495	-4702.507	-3136.801	666.4185	444.1102	1535.218	976.9417
2MG-A	1378.604	904.9279	1097.601	637.8454		-3471.365	678.727	441.9598	1536.851	982.9556
2MG-EA	1364.14	898.1331	1083.081	634.2901	-4814.303	-3182.842	674.9767	444.4344	1535.929	984.2916
2MG-CD	1381.392	906.1042	1126.402	690.2423	603.2821	380.3957	744.7802	473.3613	1542.82	989.6039
2MG-EAD	1379.392	904.1042	1124.402	688.2423	601.282	378.3956	742.7801	471.3613	1540.82	987.604
