# Supplementary figures and images for: Genetic Analyses of Flower, Fruit, and Stem Traits of Intergeneric Hybrids Between ‘Honghuagqinglong’ and ‘Heilong’ Pitayas
Source: Plants (Basel). 2024 Dec 19;13(24):3546. doi: 10.3390/plants13243546 (PMC11680067; doi:10.3390/plants13243546)

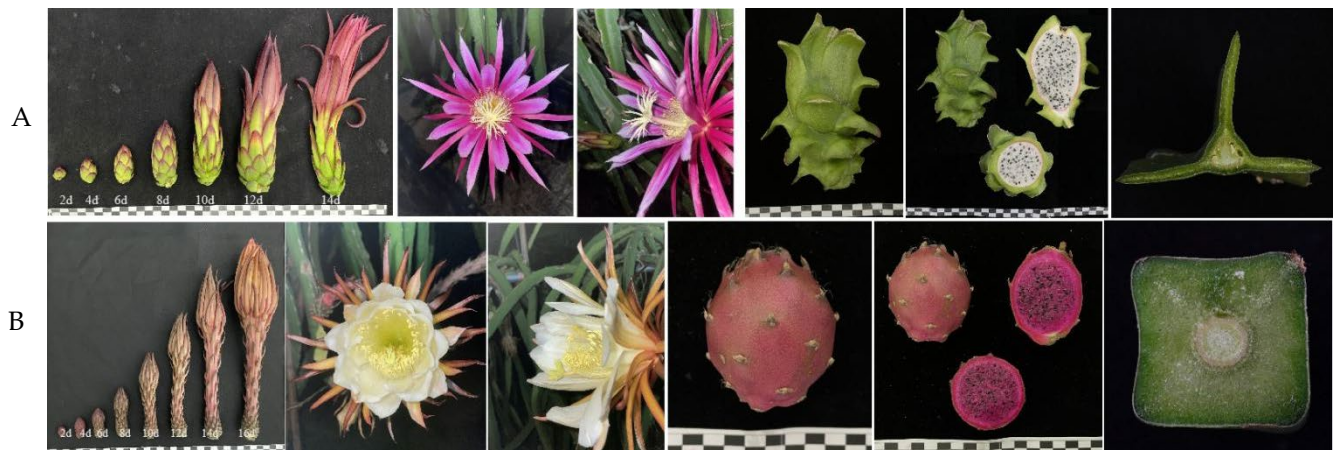

**Supplementary Figure S1.** The flower, fruit, and stem traits of 'HHQL' (A) and 'HL' (B) pitayas.

Supplement: Supplementary file 1 [file plants-13-03546-s001.zip › Supplementary Figure 1.pdf]

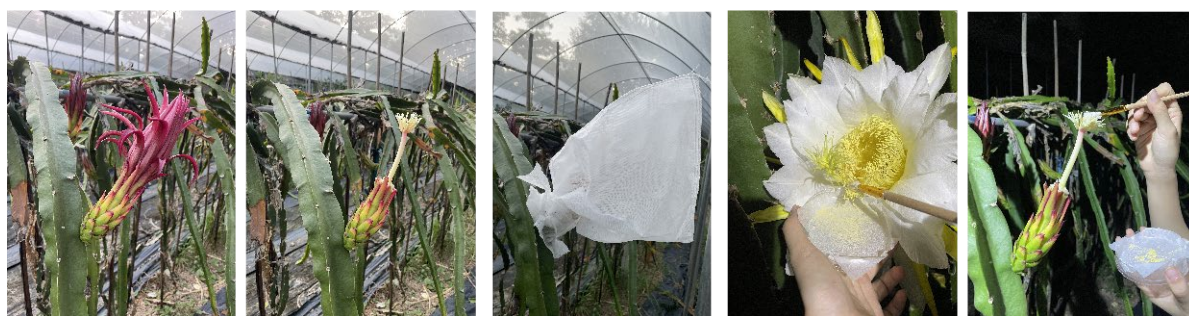

**Supplementary Figure S2** Hybridization process.

Supplement: Supplementary file 1 [file plants-13-03546-s001.zip › Supplementary Figure 2.pdf]
